# DHA elongates the neuronal primary cilium through GPR40-mediated signaling

**DOI:** 10.3389/fcell.2026.1910684

**Published:** 2026-09-29

**Authors:** Kento Karita, Mariko Yokota, Yuzuki Toyonaga, Riko Takase, Naoyuki Inagaki, Maki K. Yamada, Ikuko Yao, Manami Toriyama, Michinori Toriyama

**Affiliations:** 1 Laboratory of Systems Neurobiology and Medicine, Division of Biological Science, Nara Institute of Science and Technology, Ikoma, Japan; 2 Biomedical Engineering Major, Department of Biomedical Sciences, School of Biological and Environmental Sciences, Kwansei Gakuin University, Sanda, Japan; 3 Department of Neuropharmacology, Faculty of Pharmaceutical Sciences at Kagawa Campus, and Laboratory of Neuropharmacology in Institute of Neuroscience, Tokushima Bunri University, Takamatsu, Japan; 4 Laboratory of Neuronal Aging Regulation, Faculty of Pharmaceutical Sciences at Kagawa Campus, Institute of Neuroscience, Tokushima Bunri University, Takamatsu, Japan; 5 Laboratory of Mechano-Systems Biology and Medicine, Strategic Initiative for Research and Innovation, Nara Institute of Science and Technology, Ikoma, Japan; 6 Laboratory of Molecular Signal Transduction, Graduate School of Science and Technology, Nara Institute of Science and Technology, Ikoma, Japan

**Keywords:** ciliary length, COX-2, DHA, EP4, GPR40, NFAT, PGE2, primary cilium

## Abstract

**Introduction:**

Docosahexaenoic acid (DHA), the predominant n‐3 polyunsaturated fatty acid (PUFA) in the brain, promotes neuronal differentiation, neurite outgrowth, and synapse formation. However, the subcellular mechanisms underlying these effects remain unclear. The primary cilium functions as a signaling antenna, with its length influencing signaling capacity; however, the physiological factors regulating cilium length in neurons are largely unknown. This study investigated whether DHA promotes primary cilium elongation in neurons and examined the underlying signaling pathways.

**Methods:**

Primary cilium length was measured in cultured mouse hippocampal neurons immunostained for adenylyl cyclase 3, and in the embryonic cerebral cortex immunostained for ARL13B after maternal dietary or pharmacological manipulation of DHA and GPR40 signaling. The pathway was dissected using selective receptor agonists and antagonists, RNAi-mediated knockdown, live-cell calcium imaging, RT‐qPCR, Western blotting, and ELISA.

**Results:**

DHA increased cilium length in a concentration- and time‐dependent manner, with the increase observable within 24 h and sustained thereafter in cultured neurons. Consistently, maternal dietary n‐3 fatty acid intake modulated cilium length in the developing fetal brain, with high n‐3 diet leading to elongation and low n‐3 diet resulting in shortening. DHA-induced ciliary elongation was mediated by GPR40, as this effect was abolished by a selective GPR40 antagonist *in vitro* and recapitulated by selective GPR40 agonists both *in vitro* and *in vivo*. DHA rapidly increased intracellular Ca^2+^ levels, and pharmacological inhibition of calcineurin—NFAT signaling suppressed DHA‐induced ciliary elongation. DHA selectively induced cyclooxygenase‐2 (COX‐2) at both the mRNA and protein levels, and COX‐2 activity was essential for elongation. Finally, DHA elevated prostaglandin E2 (PGE2) levels, and pharmacological blockade of EP4 suppressed DHA‐induced ciliary elongation, indicating that PGE2–EP4 signaling contributes to this process.

**Discussion:**

By identifying the regulation of cilium length as a previously unrecognized action of DHA, this study uncovers a subcellular mechanism by which dietary n‐3 fatty acids may influence neuronal primary cilia.

## 1 Introduction

Docosahexaenoic acid (DHA, 22:6n-3) is the most abundant n-3 polyunsaturated fatty acid in the vertebrate brain, and it benefits the development and function of the central nervous system (Nguyen et al., 2014; Bazinet and Layé, 2014; Lauritzen et al., 2001). The brain accumulates DHA most rapidly during the perinatal growth spurt, when neurogenesis, neurite outgrowth, and synapse formation are at their most active (Cao et al., 2009; He et al., 2009; Kawakita et al., 2006). In the hippocampus, DHA drives neural stem cells toward a neuronal fate and expands the pool of newborn neurons (He et al., 2009; Kawakita et al., 2006), whereas maternal n-3 deficiency impairs neurogenesis in the developing brain (Coti Bertrand et al., 2006). At the synaptic level, it supports dendritic spine maturation and enhances glutamatergic transmission and long-term potentiation (Cao et al., 2009; Calderon and Kim, 2004; Sakamoto et al., 2007). In line with these roles, dietary DHA lowers amyloid burden in mouse models (Lim et al., 2005), and higher DHA intake and status are associated with a lower risk of Alzheimer’s disease (Morris et al., 2003; Schaefer et al., 2006).

Vertebrates synthesize DHA only inefficiently from dietary precursors, and the brain, which cannot make it *de novo*, depends instead on circulating DHA imported across the blood-brain barrier by the transporter MFSD2A (major facilitator superfamily domain-containing 2A). The stakes of this supply become clear when the transporter fails. Deleting Mfsd2a in mice, like pathogenic MFSD2A variants in patients, causes severe structural brain abnormalities, including microcephaly (Nguyen et al., 2014; Guemez-Gamboa et al., 2015; Alakbarzade et al., 2015). Just how DHA produces its many effects on neuronal development and function, however, remains only partly understood. One candidate mediator is the free fatty acid receptor GPR40 (G protein-coupled receptor 40; also known as free fatty acid receptor 1, FFAR1), a G protein-coupled receptor (GPCR) activated by medium and long chain free fatty acids, DHA among them. GPR40 is expressed in central neurons, notably in the primate hippocampus and its neurogenic niche (Ma et al., 2008; Yamashima, 2008), and *in situ* hybridization later placed Gpr40 mRNA in mouse hippocampal and cortical neurons, where the receptor responds to DHA (Zamarbide et al., 2014). Acting through GPR40, DHA mobilizes intracellular Ca^2+^ and promotes neuronal differentiation and neurite outgrowth (Ma et al., 2010; Yamashima, 2008). These Ca^2+^ signals are decoded in part by the calcineurin/NFAT (nuclear factor of activated T cells) pathway, a calcium-activated route to the nucleus, which places GPR40 in a position to direct calcium-dependent transcription in neurons. What links receptor activation to the actual remodeling of neuronal architecture, though, has remained largely undefined.

One neuronal structure whose signaling is organized by GPCRs, and whose remodeling might therefore fall under the same control, is the primary cilium. This solitary sensory organelle projects from the cell surface to monitor the extracellular environment. It is built around an axoneme of nine microtubule doublets, nucleated by the basal body and wrapped in a specialized membrane that concentrates GPCRs, ion channels, and growth factor receptors (Hilgendorf et al., 2024; Satir and Christensen, 2007). This enrichment lets the cilium act as a signaling antenna for pathways that direct development and tissue patterning, among them Hedgehog, Wnt, and platelet-derived growth factor receptor α (PDGFRα) (Goetz and Anderson, 2010; Anvarian et al., 2019). How much the organelle matters is evident from the ciliopathies, a group of disorders caused by defective ciliary assembly or signaling that includes Joubert, Meckel, and Bardet–Biedl syndromes (Hildebrandt et al., 2011; Reiter and Leroux, 2017). Strikingly, their neurodevelopmental features, such as agenesis of the corpus callosum, hydrocephalus, and intellectual disability, overlap with those produced by inadequate brain DHA, including the microcephaly and cognitive impairment associated with loss of MFSD2A function (Nguyen et al., 2014; Guemez-Gamboa et al., 2015; Alakbarzade et al., 2015).

In the nervous system, ciliary signaling has been tied to several aspects of hippocampal function. Sonic Hedgehog signaling relayed through the cilium drives hippocampal neurogenesis (Breunig et al., 2008). An intact cilium is required for the synaptic integration of dentate granule cells born in the adult hippocampus (Kumamoto et al., 2012). Neuronal cilia also form axo-ciliary synapses that modulate chromatin accessibility (Sheu et al., 2022). Each of these functions depends on the cilium reaching an appropriate length (Nachury and Mick, 2019), yet the physiological cues that set ciliary length in neurons remain largely undefined. One clue comes from prostaglandin E_2_ (PGE_2_), which lengthens cilia through cyclooxygenase activity and downstream signaling by the prostaglandin E receptor 4 (EP4) and cyclic adenosine monophosphate (cAMP) (Jin et al., 2014). And because Ca^2+^ acting through calcineurin and NFAT is an established inducer of cyclooxygenase-2 (COX-2) (Iñiguez et al., 2000), the GPR40/Ca^2+^ signal engaged by DHA could feed into this COX-2/PGE_2_/EP4 axis to set ciliary length, a possibility that has not yet been tested in neurons.

Here we asked whether DHA controls the length of the neuronal primary cilium, and set out to define the mechanism behind it. Combining cultured mouse hippocampal neurons with maternal dietary and pharmacological manipulations *in vivo*, we find that DHA elongates the primary cilium by acting on GPR40. Receptor activation sets off a Ca^2+^/calcineurin/NFAT cascade that induces COX-2, and the resulting PGE_2_ signals through EP4 to raise cAMP. Together these results identify ciliary length as a target of extracellular DHA and map the pathway that couples GPR40 to ciliary elongation. More broadly, they show that a dietary lipid can remodel a neuronal organelle and, in doing so, shape brain function.

## 2 Results

### 2.1 DHA promotes neuronal primary cilium elongation *in vitro*

Most central neurons extend a single primary cilium that functions as a specialized signaling compartment enriched in selected GPCRs and adenylyl cyclase type 3 (AC3, also known as ADCY3). AC3 is an enzyme concentrated in neuronal cilia and is widely used as a marker of this organelle (Berbari et al., 2007; Bishop et al., 2007). To examine whether DHA affects the neuronal primary cilium, we cultured mouse primary hippocampal neurons and visualized cilia by immunostaining for AC3. DHA was added to the medium 24 h after seeding, when neurons begin to form cilia, and ciliary length was measured 24 h later, at the second day *in vitro* (DIV2). Under these conditions, AC3-positive cilia were readily detected as distinct rod-shaped structures in both vehicle- and DHA-treated neurons (Figure 1A), and we therefore focused on ciliary length in the subsequent analyses. DHA increased ciliary length relative to vehicle in a concentration dependent manner, with a significant effect already apparent at 1 μM and a comparable, near maximal response at 2.5–10 μM (Figure 1B). To determine whether this effect was transient or sustained, neurons were treated with DHA or vehicle (DMSO), and ciliary length was measured 24 h later (DIV2) and 72 h later (DIV4). Cilia elongated over time under both conditions, consistent with the gradual maturation of neuronal cilia in culture, and DHA nonetheless increased ciliary length relative to vehicle at both time points (Figures 1C,D). The increment produced by DHA was therefore established by 24 h and maintained at 72 h, indicating that DHA did not simply accelerate elongation in a transient fashion. These data identify the neuronal primary cilium as a target of DHA, the length of which increases in a concentration dependent and time dependent manner.

**FIGURE 1:**
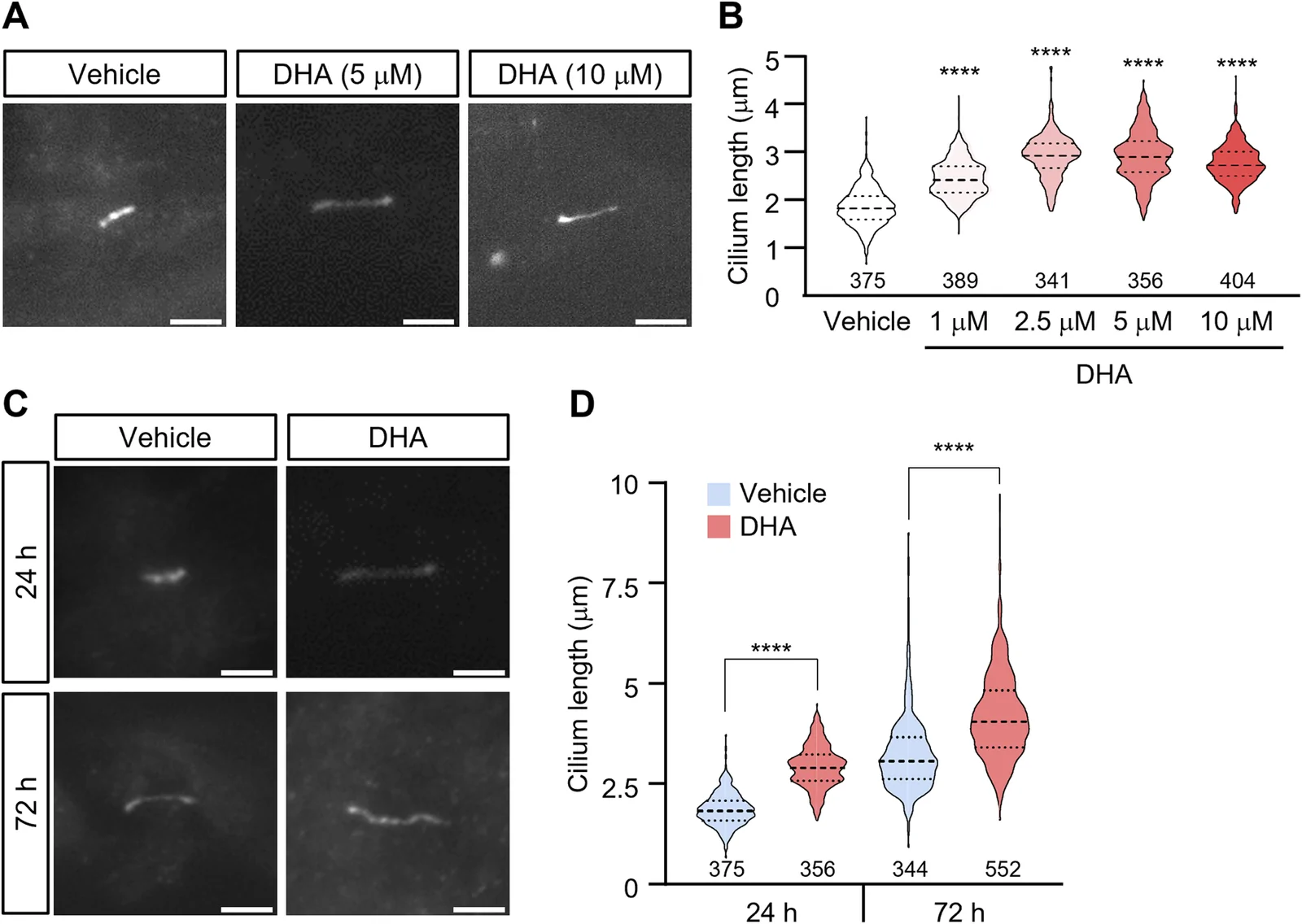
DHA promotes the elongation of primary cilia in cultured hippocampal neurons. **(A)** Representative images of primary mouse hippocampal neurons at 2 days *in vitro* (DIV2) immunostained for adenylyl cyclase 3 (AC3), a marker of neuronal primary cilia. DHA was added to the culture medium 24 h after plating, and the cells were fixed and immunostained 24 h later. Images are shown for the vehicle, 5 μM DHA, and 10 μM DHA conditions. **(B)** Quantification of primary cilium length across DHA concentrations (1, 2.5, 5, and 10 μM), showing a concentration-dependent increase relative to the vehicle. **(C)** Representative images of neurons immunostained for AC3. DHA (5 μM) or vehicle (DMSO) was added 24 h after plating, and the cells were fixed at DIV2 (24 h) and DIV4 (72 h). **(D)** Quantification of primary cilium length at each time point, showing a significant increase with DHA at both 24 and 72 h. In **(B,D)**, violin plots show the distribution of individual cilia; dashed lines indicate the median and quartiles, and the number of cilia analyzed is indicated above each violin. ****p < 0.0001 versus the corresponding vehicle control (one-way ANOVA followed by Dunnett’s test in **(B)**; two-way ANOVA followed by Šídák’s *post hoc* test in **(D)**). Scale bars, 2 μm.

### 2.2 DHA promotes neuronal primary cilium elongation *in vivo*

To ask whether DHA also promotes cilium elongation in the developing brain, we manipulated DHA availability through the maternal diet. Pregnant mice (dams) were fed a control diet (AIN-93G), an n-3-deficient diet (low n-3), or an n-3-enriched diet (high n-3) from embryonic day 7.5 (E7.5). Maternal body weight gain during the feeding period did not differ among the diet groups, and no apparent differences in fetal size or gross morphology were observed at E13.5 (Supplementary Figure S1), indicating that dietary manipulation did not affect maternal growth or overall fetal development. At E13.5, coronal sections of the fetal brain were prepared, and after immunostaining for ARL13B, the primary cilium length of the cortical neurons was measured. Cilium length in the fetal cerebral cortex varied with maternal n-3 polyunsaturated fatty acid (PUFA) intake, being longer than that in the control group in the high n-3 group and shorter than that in the control group in the low n-3 group (Figure 2 and Supplementary Figure S2). Together with the culture data, these results indicate that DHA promotes neuronal primary cilium elongation both *in vitro* and *in vivo.*

**FIGURE 2:**
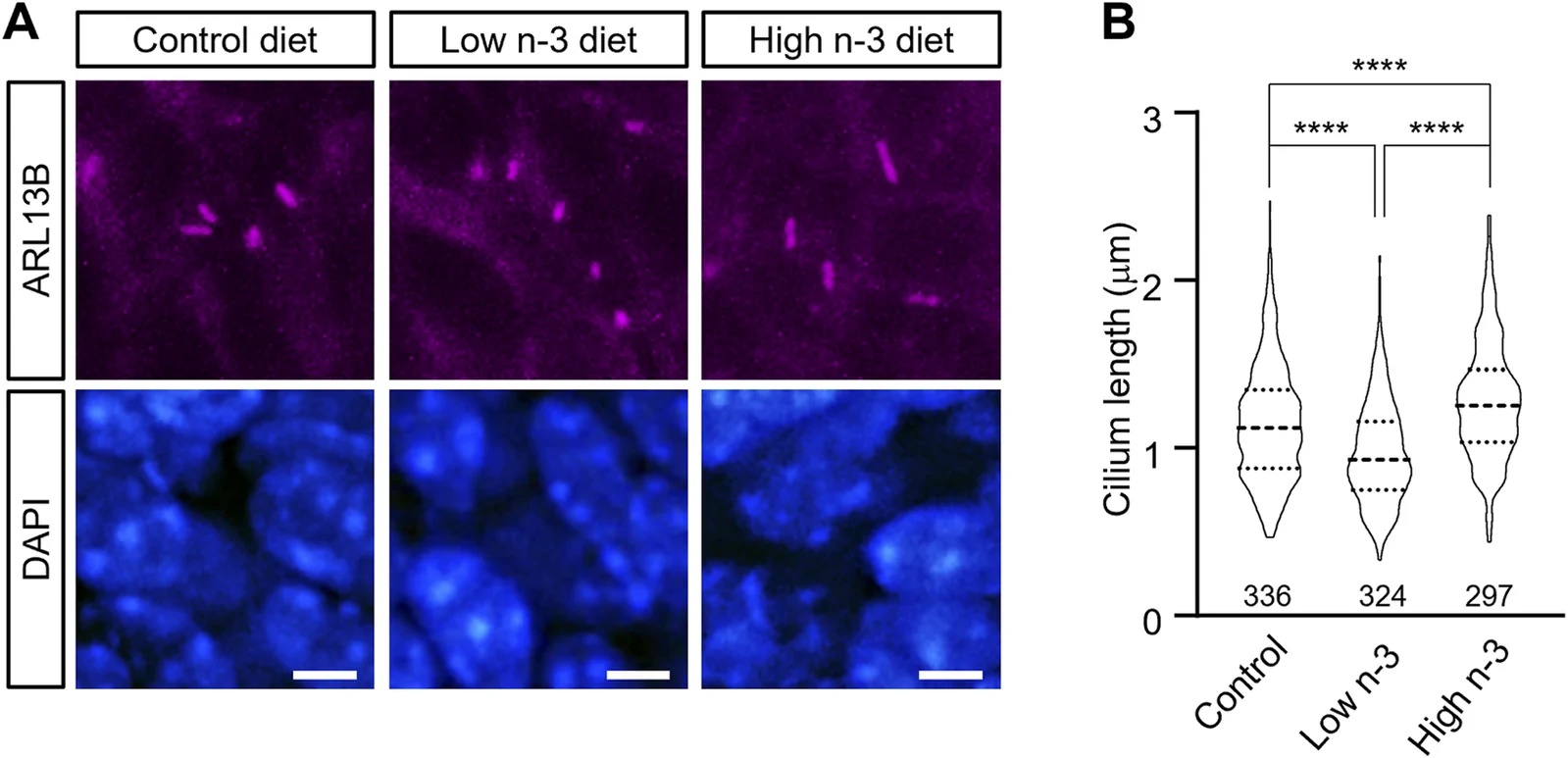
Maternal dietary n-3 PUFA intake modulates primary cilium length *in vivo*. **(A)** Pregnant dams were fed the control (AIN-93G), low-n-3, or high-n-3 diet (Table 4) from embryonic day (E) 7.5. Coronal sections of the embryonic brain were prepared at E13.5 and immunostained for the primary cilium marker ARL13B (magenta), with nuclei counterstained by DAPI (blue). Representative images of the cerebral cortex are shown. Scale bars, 10 μm. **(B)** Quantification of primary cilium length in the three dietary groups. Violin plots show the distribution of individual cilia; dashed lines indicate the median and quartiles, and the number of cilia analyzed is given above each violin. ****p < 0.0001 (one-way ANOVA followed by Tukey’s test).

### 2.3 DHA induced primary cilium elongation is mediated by GPR40

DHA is recognized by several receptors. At the plasma membrane, DHA acts as an agonist for two G protein coupled free fatty acid receptors, GPR40 (FFAR1), which is activated by medium and long chain fatty acids (Briscoe et al., 2003; Itoh et al., 2003), and GPR120 (FFAR4), a sensor of long chain unsaturated fatty acids (Hirasawa et al., 2005). As a nuclear receptor ligand, DHA also binds the peroxisome proliferator activated receptors (PPARs) to regulate transcription (Forman et al., 1997; Kliewer et al., 1997). To identify the receptor responsible for DHA-induced ciliary elongation, we first examined GPR40. Inhibition of GPR40 with the selective antagonist DC260126 abolished DHA-induced elongation and returned ciliary length to control levels, whereas DC260126 alone had no effect (Figures 3A,B). To examine whether GPR40 mediates this response *in vivo*, we administered DHA to pregnant mice with or without DC260126 and examined the cortical neuron cilia at E13.5. Maternal administration of DHA increased ciliary length, and co-administration of DC260126 abolished this increase (Figures 3C,D). To validate these pharmacological findings using an independent genetic approach, we knocked down endogenous GPR40 in cultured hippocampal neurons using two independent RNAi constructs directed against non-overlapping regions of the mouse *Ffar1*. Because each vector co-expresses EmGFP from the same transcript, transfected neurons were identified by EmGFP fluorescence, allowing the ciliary length to be assessed specifically in cells expressing the construct. Knockdown efficiency was verified in HEK293T cells co-expressing GPR40-EGFP, in which both constructs markedly reduced GPR40-EGFP levels (Figures 3E,F). Neurons were transfected immediately after dissociation and treated with DHA from DIV1 for 24 h. DHA increased ciliary length as expected in control-RNAi treated neurons, whereas this increase was abolished in neurons expressing either GPR40-RNAi construct, and ciliary length in these neurons remained comparable to that of their vehicle-treated counterparts (Figures 3G,H). Thus, DHA-induced primary cilium elongation was abolished by both pharmacological inhibition and genetic knockdown of GPR40, demonstrating that GPR40 is required for cilium elongation in hippocampal neurons.

**FIGURE 3:**
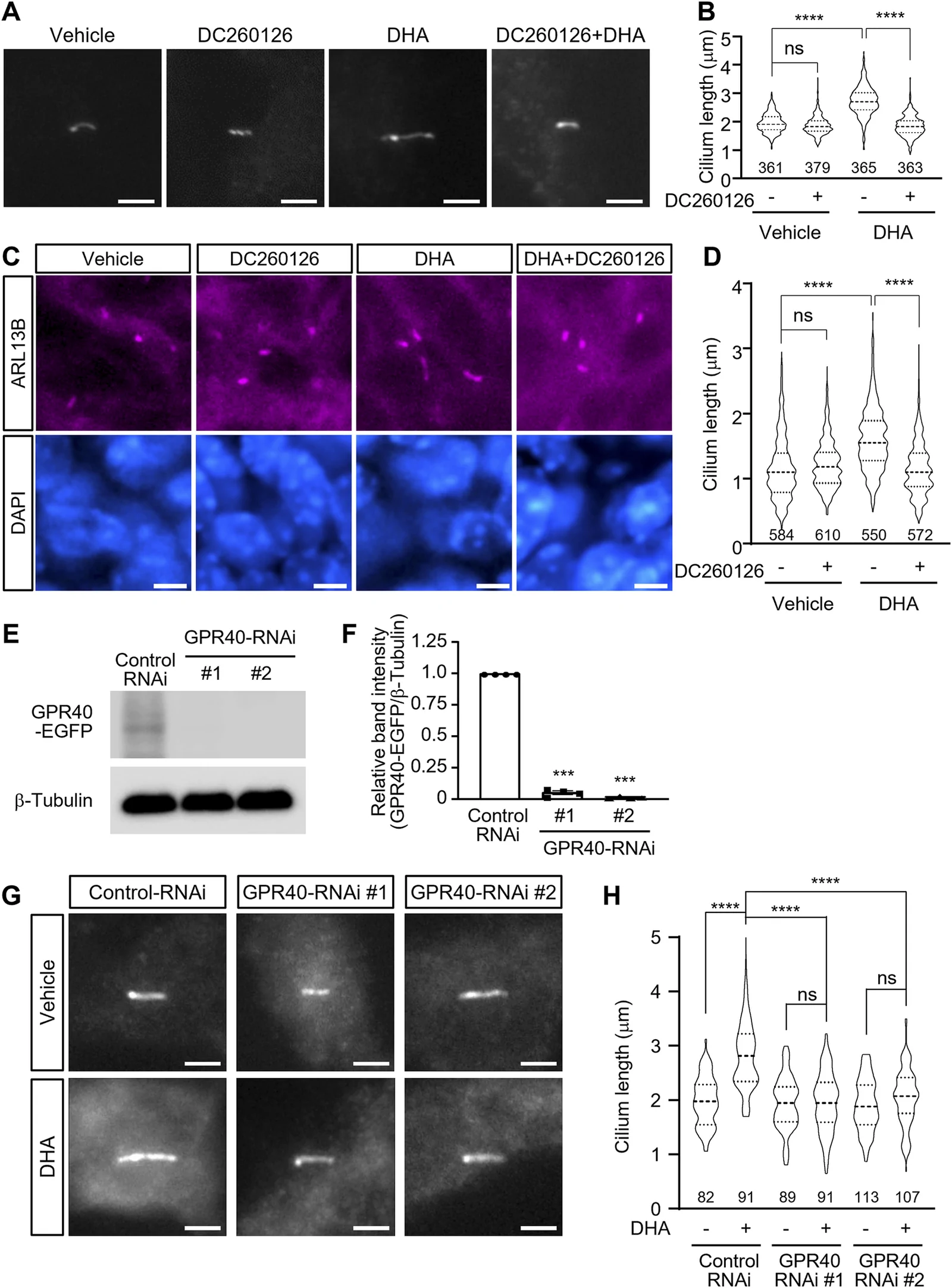
DHA induced primary cilium elongation is mediated by GPR40 both *in vitro* and *in vivo*. **(A)** Representative images of primary mouse hippocampal neurons at DIV2 immunostained for AC3, treated with vehicle or DHA (5 μM) in the absence or presence of the GPR40 antagonist DC260126 (10 μM). Scale bars, 2 μm. **(B)** Quantification of cilium length in (A). DHA increased cilium length, and this increase was abolished by DC260126, whereas DC260126 alone had no effect. **(C)** Pregnant dams were administered DHA and/or DC260126 (50 mg/kg each, intraperitoneally) on embryonic day (E) 11.5. Embryonic brains were collected at E13.5, and coronal sections were immunostained for the ciliary marker ARL13B (magenta), with nuclei counterstained by DAPI (blue). Primary cilia in the preplate of the cerebral cortex were analyzed. Scale bars, 5 μm. **(D)** Quantification of cilium length in **(C)**. Maternal administration of DHA increased cilium length in cortical neurons, and co administration of DC260126 abolished this increase. **(E,F)** Validation of knockdown efficiency. HEK293T cells were co transfected with GPR40-EGFP and either a non-targeting control vector (Control-RNAi) or one of two independent RNAi vectors targeting mouse *Ffar1* (GPR40-RNAi #1 and #2). Lysates were collected 48 h after transfection and immunoblotted for GFP and β-tubulin. **(E)** Representative immunoblots. **(F)** Quantification of GPR40-EGFP band intensity normalized to β-tubulin and expressed relative to the control. Both constructs markedly reduced GPR40-EGFP expression. Data are presented as mean ± SEM (n = 4 independent cultures). ***p < 0.001 versus Control-RNAi. **(G)** Primary mouse hippocampal neurons were transfected with the indicated RNAi vectors immediately after dissociation, treated with vehicle (DMSO) or DHA (5 μM) from DIV1 for 24 h, and fixed at DIV2. Cilium length was measured in EmGFP positive neurons immunostained for AC3. Scale bars, 2 μm. **(H)** Quantification of cilium length in (G). DHA increased cilium length in control neurons, whereas no such increase was observed with either GPR40-RNAi construct; cilium length in DHA treated GPR40-RNAi neurons was significantly shorter than in DHA treated control-RNAi neurons. Neither construct altered cilium length under vehicle conditions. In **(B,D,H)**, violin plots show the distribution of individual cilia, with dashed lines indicating the median and quartiles; the number of cilia analyzed is given above each violin. ****p < 0.0001; ns, not significant (two-way ANOVA followed by Tukey’s *post hoc* test).

We next asked whether activation of GPR40 is sufficient for ciliary elongation. Treatment with the selective GPR40 agonists TAK-875 or GW9508 in the absence of DHA increased ciliary length and reproduced the effect of DHA (Figures 4A,B). Consistently, maternal administration of GW9508 to pregnant mice increased ciliary length in cortical neurons at E13.5 (Figures 4C,D), reproducing the *in vitro* findings in the brain. Because DHA is also a ligand for nuclear PPARs, we examined whether PPARγ contributes to ciliary elongation. Before the addition of DHA, neurons were pretreated with the selective PPARγ antagonists G3335 or GW9662. Neither antagonist altered ciliary length on its own, and DHA increased ciliary length to a comparable extent in their presence (Supplementary Figure S3), indicating that PPARγ is not required. Taken together, these loss of function and gain of function experiments performed *in vitro* and *in vivo* show that DHA induced primary cilium elongation depends on GPR40 but not on PPARγ. This is consistent with a model in which extracellular DHA is sensed by GPR40 at the plasma membrane and elongates the cilium through downstream intracellular signaling.

**FIGURE 4:**
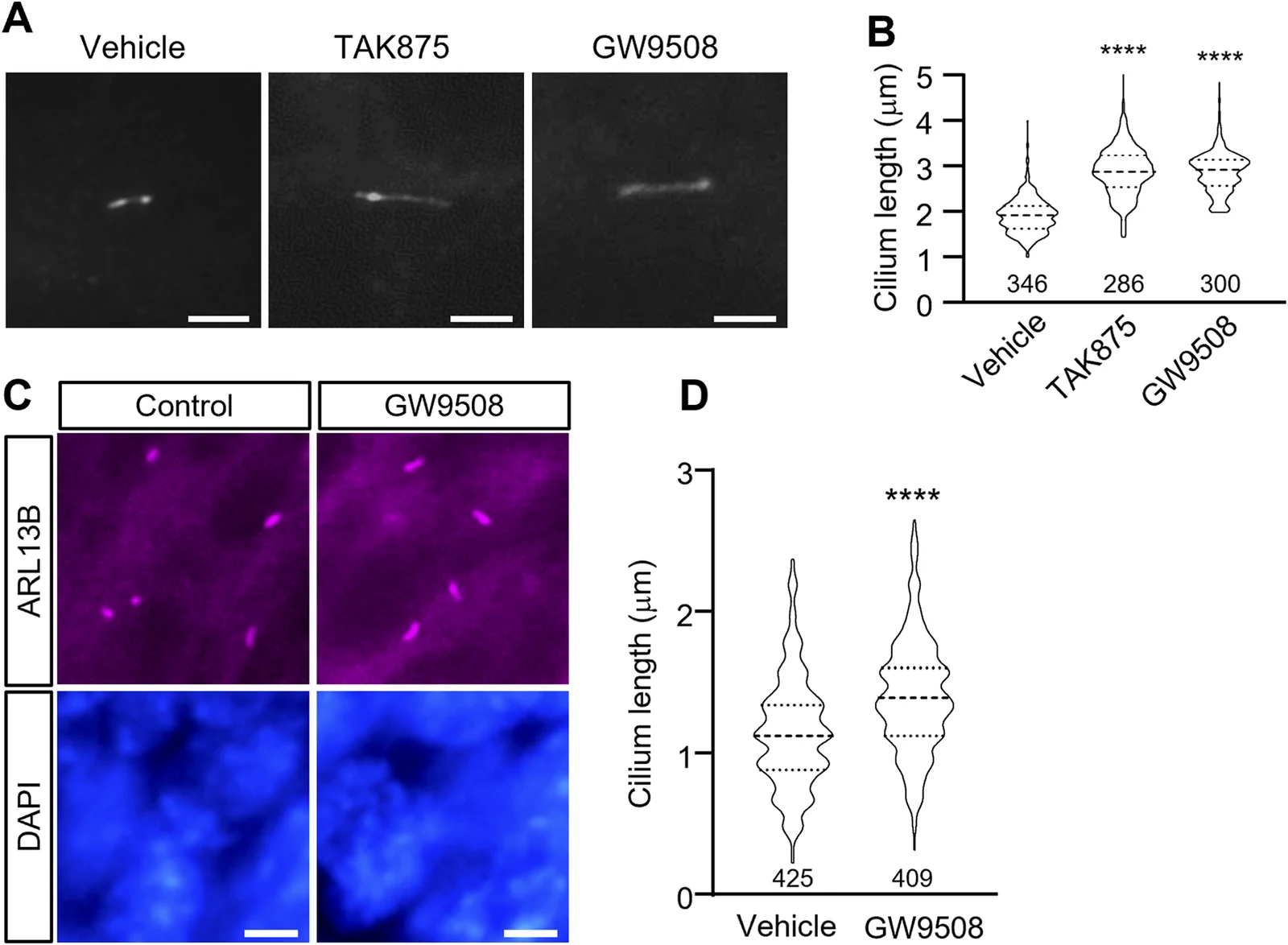
Activation of GPR40 is sufficient to elongate primary cilia both *in vitro* and *in vivo*. **(A)** Representative images of neurons treated with the GPR40 agonists TAK-875 (25 μM) or GW9508 (25 μM) and immunostained for AC3. Scale bars, 2 μm. **(B)** Quantification of primary cilium length *in vitro* after treatment with TAK-875 and GW9508 at 25 μM. **(C)** Pregnant dams were administered the GPR40 agonist GW9508 (50 mg/kg, intraperitoneally) at E11.5, and the embryonic brains were collected at E13.5. Coronal sections were immunostained for ARL13B (magenta) and counterstained with DAPI (blue). Primary cilia in the preplate of the cerebral cortex were analyzed. Scale bars, 5 μm. **(D)** Quantification of primary cilium length, showing that maternal GW9508 administration increased ciliary length. In all violin plots, dashed lines indicate the median and quartiles, and the number of cilia analyzed is given above each violin. ****p < 0.0001. Statistical tests: one-way ANOVA with Dunnett’s test **(B)**; Mann–Whitney test **(D)**.

### 2.4 GPR40 promotes ciliary elongation through Ca^2+^ signaling and the phosphatase calcineurin

Having identified GPR40 as the receptor responsible for DHA induced elongation, we examined the downstream signaling. In pancreatic β cells, GPR40 couples to Gq and raises intracellular Ca^2+^ (Briscoe et al., 2003; Itoh et al., 2003; Mancini and Poitout, 2013). To examine whether GPR40 mobilizes Ca^2+^ in neurons, we expressed the Ca^2+^ sensor jRGECO1a in cultured hippocampal neurons and monitored fluorescence by live imaging. DHA evoked a rapid rise in intracellular Ca^2+^ (Figure 5A), showing that DHA raises intracellular Ca^2+^ in hippocampal neurons.

**FIGURE 5:**
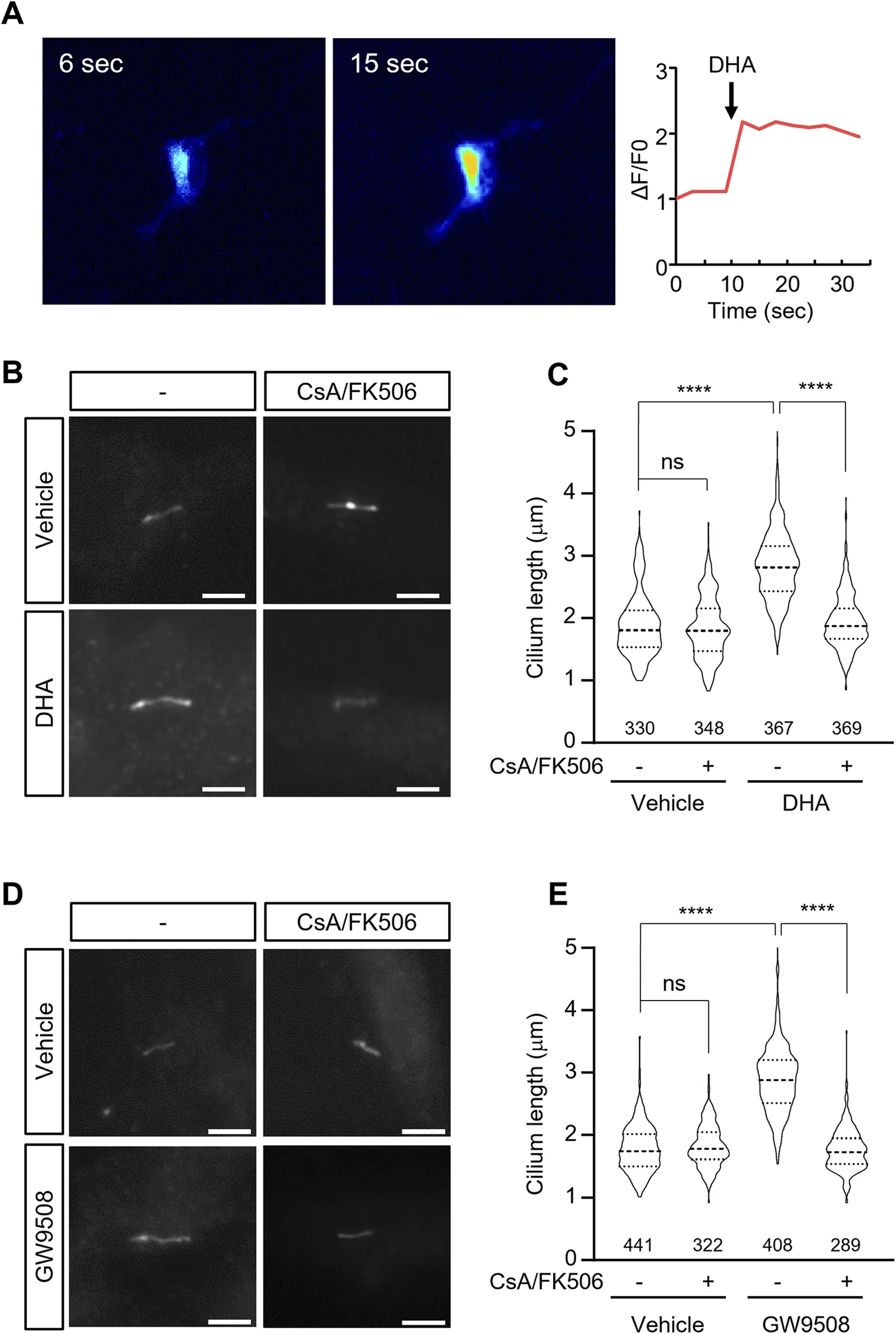
DHA induces an intracellular Ca^2+^ increase that drives calcineurin-dependent primary cilium elongation. **(A)** Representative pseudocolored images (left) and a representative trace (right) of intracellular Ca^2+^ in cultured hippocampal neurons (DIV2) expressing the Ca^2+^ sensor jRGECO1a, before (6 s) and after (15 s) DHA application. Warmer colors indicate higher Ca^2+^ levels. DHA was administered 10 s after the start of observation. Scale bar, 20 μm. **(B)** Representative images of neurons (DIV2) immunostained for AC3 and treated with vehicle or DHA (5 μM) in the absence or presence of the calcineurin inhibitors cyclosporine A (CsA, 200 nM) and FK-506 (10 μM) (CsA/FK-506). Scale bars, 2 μm. **(C)** Quantification of primary cilium length after treatment with 5 μM DHA, 200 nM CsA and/or 10 μM FK-506. **(D)** Representative images of neurons (DIV2) immunostained for AC3 and treated with vehicle or the GPR40 agonist GW9508 (25 μM) in the absence or presence of CsA/FK-506. Scale bars, 2 μm. **(E)** Quantification of primary cilium length after treatment with 25 μM GW9508, 200 nM CsA and/or 10 μM FK-506. In **(C,E)**, violin plots show the distribution of individual cilia; dashed lines indicate the median and quartiles, and the number of cilia analyzed is indicated above each violin. ****p < 0.0001; ns, not significant (two-way ANOVA followed by Tukey’s *post hoc* test; N = 3 independent cultures).

Because Ca^2+^ acts mainly through Ca^2+^ binding effectors, we focused on calcineurin, a Ca^2+^/calmodulin dependent serine/threonine phosphatase that is abundant in neurons. To examine whether calcineurin is required for ciliary elongation, we treated neurons with the calcineurin inhibitors cyclosporine A and FK-506 (CsA/FK-506). DHA increased ciliary length relative to control at DIV2, whereas co-treatment with CsA/FK-506 abolished this effect, and the inhibitors alone produced no significant change (Figures 5B,C). The elongation induced by the GPR40 agonist GW9508 was likewise abolished by CsA/FK-506 (Figures 5D,E). Calcineurin dephosphorylates substrates that govern neurite outgrowth, gene expression, synaptic plasticity, and learning and memory (Hoffman et al., 2017). These results indicate that GPR40, activated by DHA, raises intracellular Ca^2+^ and promotes primary cilium elongation through calcineurin.

### 2.5 Activation of the calcineurin dependent transcription factor NFAT mediates ciliary elongation

Because calcineurin acts on many substrates, we sought the effector that links it to ciliary elongation. We focused on the nuclear factor of activated T cells (NFAT) family for two reasons. First, NFAT proteins are canonical direct substrates of calcineurin and represent a major route by which Ca^2+^/calcineurin signals reach the nucleus. Second, the elongation we observed developed gradually and persisted over several days (Figures 1C,D), pointing to a transcriptional program rather than acute post-translational regulation. In neurons, including cultured hippocampal neurons, activity dependent Ca^2+^ elevation activates calcineurin and promotes the dephosphorylation and nuclear translocation of NFAT (Ulrich et al., 2012).

Of the five NFAT proteins (NFAT1 to NFAT5; Crabtree and Olson, 2002), NFAT1 to NFAT4 are regulated by intracellular Ca^2+^ through calcineurin. In the resting state, NFAT is phosphorylated and retained in the cytoplasm, but a rise in intracellular Ca^2+^ activates calcineurin, which dephosphorylates NFAT, exposes its nuclear localization signal, and allows nuclear translocation and transcription of target genes (Hogan et al., 2003). NFATc3 translocates to the nucleus relatively rapidly in hippocampal and sensory neurons (Ulrich et al., 2012), and NFAT activity has been linked to COX-2 expression in sensory neurons (Jackson et al., 2007; Groth et al., 2007), suggesting a transcriptional output through which NFAT could influence ciliary length.

To examine whether the calcineurin/NFAT axis is required for DHA induced elongation, we used INCA-6, which selectively disrupts the calcineurin/NFAT interaction without broadly inhibiting the phosphatase activity of calcineurin, thereby preventing the dephosphorylation of NFAT by calcineurin. DHA increased ciliary length at DIV2, and INCA-6 abolished this increase (Figures 6A,B). The elongation induced by GW9508 was likewise abolished by INCA-6, whereas INCA-6 alone had no effect (Figures 6C,D). These results show that NFAT activity is required for DHA and GPR40 induced primary cilium elongation, consistent with a model in which Ca^2+^ activated calcineurin dephosphorylates NFAT and NFAT dependent transcription drives elongation.

**FIGURE 6:**
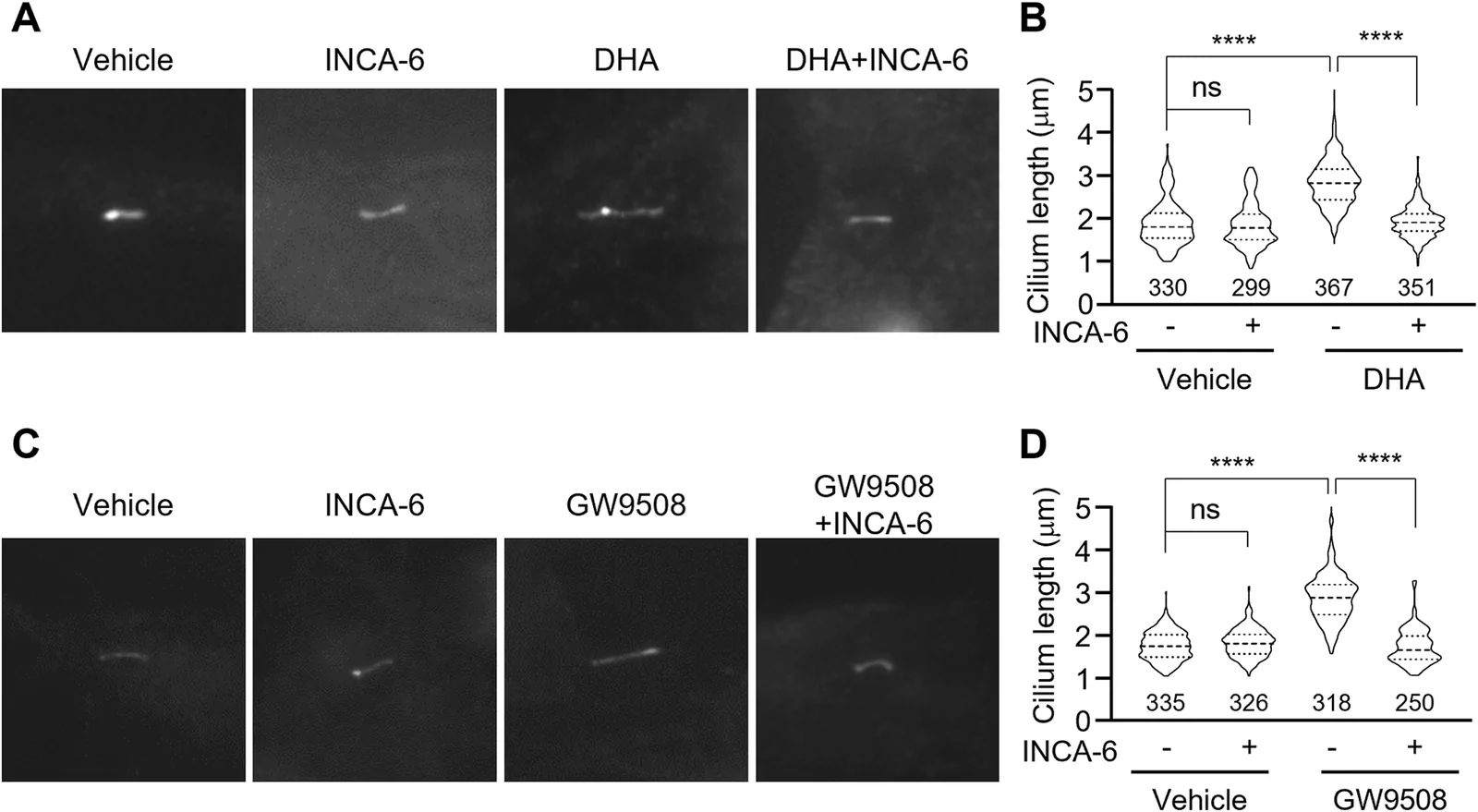
DHA- and GPR40-induced primary cilium elongation requires calcineurin–NFAT signaling. **(A)** Representative images of primary mouse hippocampal neurons at 2 days *in vitro* (DIV2) immunostained for AC3, treated with vehicle or DHA (5 μM) in the absence or presence of INCA-6 (1 μM), an inhibitor of the calcineurin–NFAT interaction. Scale bars, 2 μm. **(B)** Quantification of primary cilium length after treatment of 5 μM DHA and 1 μM INCA-6. **(C)** Representative images of neurons (DIV2) immunostained for AC3, treated with vehicle or the GPR40 agonist GW9508 (25 μM) in the absence or presence of INCA-6 (1 μM). Scale bars, 2 μm. **(D)** Quantification of primary cilium length after treatment of 25 μM GW9508 and 1 μM INCA-6. In all violin plots **(B,D)**, dashed lines indicate the median and quartiles, and the number of cilia analyzed is given above each violin. ****p < 0.0001; ns, not significant (two-way ANOVA followed by Tukey’s *post hoc* test; N = 3 independent cultures).

### 2.6 DHA selectively induces COX-2 expression, and COX-2 activity is required for DHA and GPR40 induced ciliary elongation

NFAT has been most extensively studied in immune cells, where its target genes include interleukins and COX-2 (Iñiguez et al., 2000; Jackson et al., 2007; Groth et al., 2007). COX-2 converts arachidonic acid to PGE_2_, which is secreted and acts in an autocrine and paracrine manner. PGE_2_ has been reported to promote ciliary elongation (Jin et al., 2014). We therefore hypothesized that activation of NFAT downstream of DHA and GPR40 induces COX-2 and thereby promotes elongation. The two cyclooxygenase isoforms differ in their mode of regulation. COX-1 is constitutively expressed in most tissues, whereas COX-2 is an inducible enzyme expressed transiently in response to hormonal and inflammatory stimuli (Sirois et al., 1992). To examine whether DHA regulates these isoforms differentially, we first measured COX-1 (*Ptgs1*) and COX-2 (*Ptgs2*) mRNA by quantitative RT-PCR after DHA treatment. COX-2 mRNA was upregulated markedly at 12 h, whereas COX-1 mRNA showed no significant change (Figure 7A). DHA therefore selectively raised COX-2 at the transcriptional level. Consistent with the mRNA data, Western blot analysis revealed a marked increase in COX-2 protein in cultured neurons treated with DHA or with the GPR40 agonists TAK-875 or GW9508 (Figures 7B,C). Because the agonists reproduced the effect of DHA, COX-2 induction lies downstream of GPR40.

**FIGURE 7:**
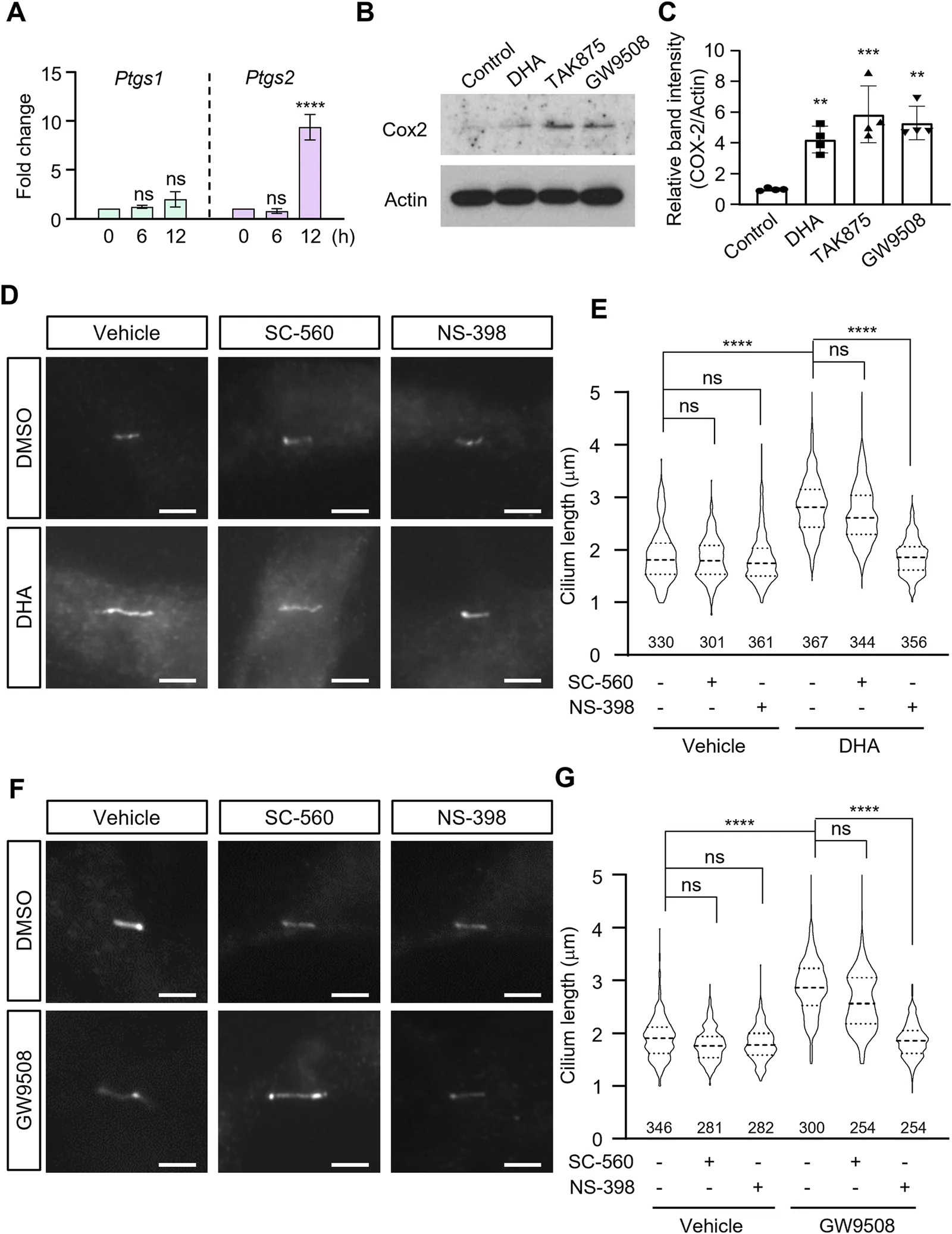
DHA-induced primary cilium elongation is mediated by COX-2. **(A)** Quantitative real-time RT-PCR analysis of *Ptgs1* (COX-1) and *Ptgs2* (COX-2) mRNA in cultured hippocampal neurons (DIV2) treated with DHA (5 μM) for 0, 6, or 12 h. *Ptgs2*, but not *Ptgs1*, was significantly upregulated 12 h after DHA treatment. Data are presented as fold change relative to 0 h (mean ± SEM). **(B)** Western blot analysis of COX-2 in neurons (DIV2) treated for 12 h with DHA (5 μM), TAK-875 (25 μM), or GW9508 (25 μM), with actin as a loading control. **(C)** Quantification of COX-2 band intensity normalized to actin (mean ± SEM; N = 4 independent cultures). **(D)** Representative images of neurons (DIV2) immunostained for AC3, treated with vehicle (DMSO) or DHA (5 μM) in the absence or presence of the COX-1 inhibitor SC-560 (5 μM) or the COX-2 inhibitor NS-398 (5 μM). Scale bars, 2 μm. **(E)** Quantification of primary cilium length after treatment of 5 μM DHA, 5 μM SC-560 and/or 5 μM NS-398. **(F)** Representative images of neurons (DIV2) immunostained for AC3, treated with vehicle or the GPR40 agonist GW9508 (25 μM) in the absence or presence of SC-560 (5 μM) or NS-398 (5 μM). Scale bars, 2 μm. **(G)** Quantification of primary cilium length after treatment of 25 μM GW9508, 5 μM SC-560 and/or 5 μM NS-398. In the violin plots **(E,G)**, dashed lines indicate the median and quartiles, and the number of cilia analyzed is given above each violin. In **(A)**, ****p < 0.0001; in **(C)**, **p < 0.01 and ***p < 0.001; in **(E,G)**, ****p < 0.0001; ns, not significant (one-way ANOVA followed by Dunnett’s test in A and C; two-way ANOVA followed by Tukey’s *post hoc* test in **E,G**).

To determine which isoform mediates elongation, we treated neurons with the COX-1 selective inhibitor SC-560 or the COX-2 selective inhibitor NS-398 prior to DHA and analyzed ciliary length in neurons. SC-560 did not prevent DHA induced cilium elongation, whereas NS-398 abolished it (Figures 7D,E), indicating that COX-2 activity, but not COX-1 activity, is required. The same result was obtained when GW9508 was used in place of DHA. SC-560 did not prevent GW9508 induced elongation, whereas NS-398 abolished it (Figures 7F,G). COX-2 activity is therefore required for both DHA and GPR40 induced elongation. Taken together, DHA selectively induces COX-2 at the mRNA and protein levels through GPR40, and the resulting elongation requires COX-2 activity, consistent with a model in which GPR40 driven, NFAT dependent COX-2 induction promotes elongation through PGE_2_.

### 2.7 DHA induced primary cilium elongation is mediated by COX-2 derived PGE_2_ acting through EP4

Having shown that COX-2 activity is required for DHA induced elongation, we next turned to the prostaglandin signaling that lies downstream of this enzyme. COX-2 converts arachidonic acid to PGE_2_, which is secreted and acts through four E-prostanoid receptors, EP1 to EP4 (Sugimoto and Narumiya, 2007). To examine whether DHA activates a COX-2 dependent prostaglandin pathway, we measured PGE_2_ levels in the culture medium of hippocampal neurons. DHA significantly increased PGE_2_ levels at both 12 h and 24 h compared with baseline (Figure 8A), consistent with the observed DHA induced increase in COX-2 expression.

**FIGURE 8:**
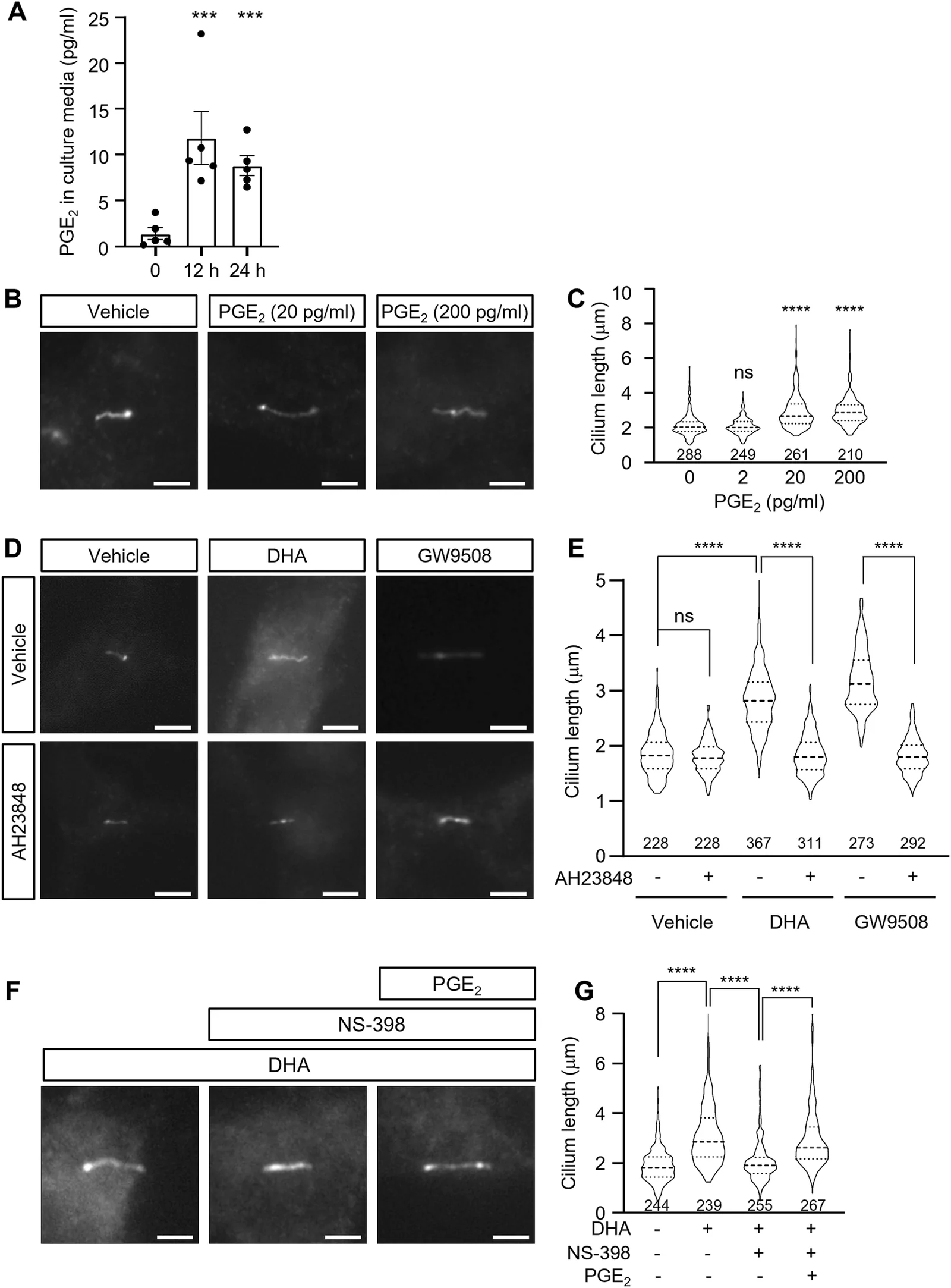
DHA induced primary cilium elongation is mediated by COX-2 derived PGE_2_ acting through the EP4 receptor. **(A)** PGE_2_ concentration in the culture medium of hippocampal neurons (DIV2) treated with DHA (5 μM) for 0, 12, or 24 h, measured by ELISA. DHA increased PGE_2_ secretion. Data are presented as mean ± SEM. ***p < 0.001 versus 0 h (one-way ANOVA followed by Dunnett’s test; N = 5 independent cultures). **(B)** Representative images of neurons (DIV2) immunostained for AC3 after treatment with vehicle or PGE_2_ (20 or 200 pg/mL). Scale bars, 2 μm. **(C)** Quantification of cilium length across PGE_2_ concentrations (0, 2, 20, and 200 pg/mL). PGE_2_ increased cilium length at 20 and 200 pg/mL. **(D)** Representative images of neurons (DIV2) immunostained for AC3 after treatment with vehicle, DHA (5 μM), or the GPR40 agonist GW9508 (25 μM) in the absence or presence of the EP4 receptor antagonist AH23848 (10 μM). Scale bars, 2 μm. **(E)** Quantification of cilium length in **(D)**. The increase in cilium length induced by either DHA or GW9508 was abolished by AH23848, whereas AH23848 alone had no effect. **(F)** Representative images of neurons (DIV2) immunostained for AC3. Neurons were treated with vehicle or DHA (5 μM) in the absence or presence of the selective COX-2 inhibitor NS-398 (5 μM), and PGE_2_ (20 pg/mL) was added to NS-398 treated cultures. Scale bars, 2 μm. **(G)** Quantification of cilium length in **(F)**. DHA increased cilium length, and this increase was abolished by NS-398. Addition of exogenous PGE_2_ restored cilium length in the presence of NS-398. In **(C,E,G)**, violin plots show the distribution of individual cilia, with dashed lines indicating the median and quartiles; the number of cilia analyzed is given above each violin. ****p < 0.0001; ns, not significant (one-way ANOVA followed by Dunnett’s test in **(C)**; two-way ANOVA followed by Tukey’s *post hoc* test in **(E)**; one-way ANOVA followed by Tukey’s *post hoc* test in **(G)**; N = 3 independent cultures).

As DHA elevated PGE_2_ in cultured neurons, we next asked whether PGE_2_ is itself sufficient to elongate the neuronal primary cilium. We treated neurons with PGE_2_ over a range of concentrations and measured ciliary length. Ciliary length was unchanged at 2 pg/mL relative to vehicle, whereas it increased significantly at 20 pg/mL and 200 pg/mL, with comparable responses at the two higher concentrations (Figures 8B,C). Because 20 pg/mL lies within the range of the PGE_2_ levels secreted by DHA treated neurons (Figure 8A), the amount of PGE_2_ produced in response to DHA is sufficient to account for the elongation.

Among the E-prostanoid receptors, EP4 localizes to the ciliary membrane, where binding of PGE_2_ activates ciliary adenylyl cyclase to raise cAMP and, through downstream PKA activation, accelerates anterograde intraflagellar transport (IFT) (Jin et al., 2014). To examine whether this PGE_2_ and EP4 axis mediates the neuronal response, we treated neurons with DHA in the absence or presence of the selective EP4 antagonist AH23848 and measured ciliary length at DIV2. Both DHA and GW9508 increased ciliary length, and AH23848 abolished both increases, whereas the antagonist alone had no effect (Figures 8D,E). Signaling through PGE_2_ and its receptor EP4 is therefore required for both DHA and GPR40 induced elongation.

To directly test whether PGE_2_ is the mediator supplied by COX-2, we investigated whether exogenous PGE_2_ could restore elongation under COX-2 inhibition. As before, NS-398 abolished the DHA-induced increase in ciliary length, and the addition of exogenous PGE_2_ to NS-398 treated cultures restored ciliary length to a level comparable to that of neurons treated with DHA alone (Figures 8F,G). Therefore, the loss of elongation under COX-2 inhibition is attributable to the reduced availability of PGE_2_ rather than to an unrelated action of the inhibitor, placing PGE_2_ downstream of COX-2 and upstream of EP4.

EP4 is a Gαs-coupled receptor that raises intracellular cAMP levels through adenylyl cyclase, and cAMP is an established determinant of ciliary length in other systems (Ou et al., 2009; Besschetnova et al., 2010). To examine whether adenylyl cyclase activity was required, we treated the neurons with a non-selective adenylyl cyclase inhibitor, SQ22536. SQ22536 abolished the DHA-induced increase in ciliary length, whereas the inhibitor alone had no effect on the basal ciliary length (Supplementary Figure S4). Together, these results place COX-2 derived PGE_2_, EP4, and adenylyl cyclase-dependent cAMP production downstream of DHA and GPR40 in the control of primary neuronal cilium length.

## 3 Discussion

Here, we show that DHA promotes primary cilium elongation in neurons. In vehicle-treated cultures, cilia elongated gradually from 24 h (DIV2) to 72 h (DIV4), and DHA increased ciliary length beyond this baseline at both time points. Cortical neuron cilia normally elongate over the first postnatal weeks (Arellano et al., 2012), and cultured neurons form cilia as they mature (Berbari et al., 2007). Therefore, the elongation in our control cultures likely reflects intrinsic maturation. Accordingly, we interpret the effect of DHA not as aberrant *de novo* elongation but as an acceleration of an already ongoing developmental elongation.

These findings are consistent with the pathways defined in this study. Ciliary length reflects the balance between the assembly and disassembly of the axoneme at the ciliary tip, governed by intraflagellar transport (IFT), and cAMP signaling regulates cilium elongation in mammals (Ou et al., 2009; Besschetnova et al., 2010). We found that DHA, acting through GPR40, drives calcineurin- and NFAT-dependent COX-2 induction and PGE_2_ production, and that PGE_2_ acts on ciliary EP4 receptors to promote elongation (Figure 9). This closely corresponds to the prostaglandin-dependent cascade reported in other systems, in which PGE_2_ binds EP4 to raise cAMP and accelerate anterograde IFT (Jin et al., 2014; Jin and Zhong, 2022). Consistent with this, blockade of EP4 with AH23848 abolished both DHA and GPR40 induced elongation (Figures 8D,E), and inhibition of adenylyl cyclase with SQ22536 likewise abolished the DHA response (Supplementary Figure S4), consistent with reports that raising cAMP is sufficient to elongate cilia in non-neuronal cells (Besschetnova et al., 2010).

**FIGURE 9:**
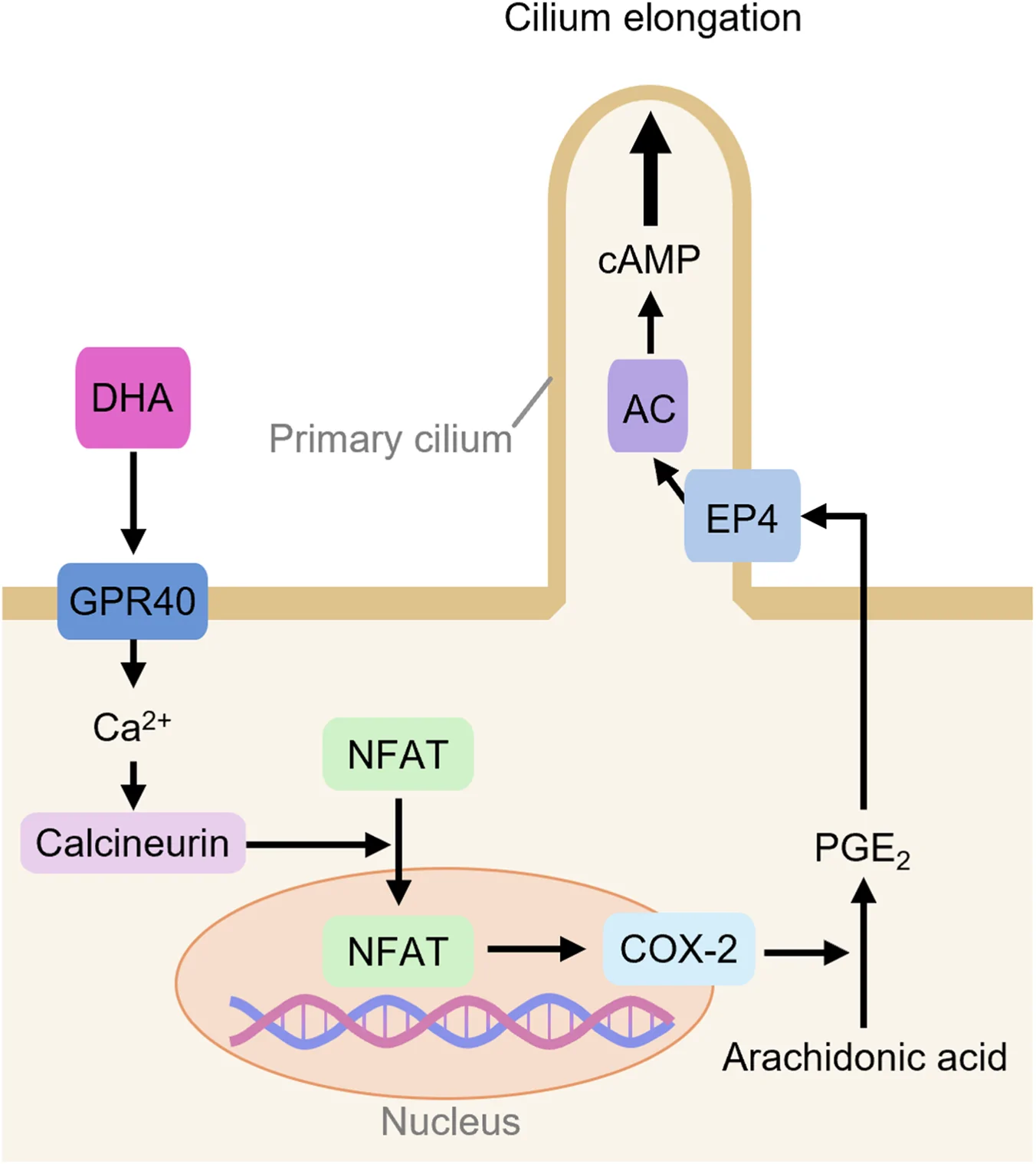
Proposed model for DHA-induced primary cilium elongation in neurons. Schematic model summarizing the findings of this study. Extracellular DHA is recognized by GPR40 at the plasma membrane, triggering an intracellular increase in Ca^2+^ levels, which subsequently activates calcineurin. Calcineurin dephosphorylates NFAT, enabling its translocation to the nucleus, where it induces the expression of COX-2. COX-2 converts arachidonic acid into PGE_2_, which acts on the ciliary EP4 receptor to increase cAMP levels via adenylyl cyclase. This sequence of events ultimately promotes primary cilium elongation.

One difference concerns the cyclooxygenase isoforms. Jin et al. (2014) found that COX-1, rather than COX-2, sets ciliary length in the zebrafish Kupffer’s vesicle, even though both isoforms can supply PGE_2_. In contrast, in our system, cilium elongation downstream of DHA and GPR40 required COX-2 and not COX-1 (Figures 7D–G), indicating that COX-2 generates PGE_2_ that drives elongation in neurons. A comparable regulatory relationship has been described in vascular endothelial cells, where the arachidonic acid epoxide 11,12-EET acts through GPR40 to increase COX-2 expression (Park et al., 2018); our results extend this relationship to neurons. Because both calcineurin/NFAT and COX-2 activities are required for ciliary elongation, we propose that COX-2 induction lies downstream of calcineurin/NFAT signaling in neurons. This induction proceeds through ERK-dependent signaling in endothelial cells (Park et al., 2018), whereas our data implicate calcineurin and NFAT in neurons; the transcription factor acting directly at the *Ptgs2* promoter, however, remains to be defined. The conclusions drawn here are based on converging evidence from independent tools at each step of the pathway. Two structurally unrelated GPR40 agonists, TAK-875 and GW9508, each reproduced the effect of DHA, and DHA-induced elongation was abolished both by pharmacological blockade with DC260126 and by genetic knockdown of the receptor. Downstream, calcineurin was inhibited by two agents acting through distinct immunophilins (cyclosporine A and FK-506), and the cyclooxygenase requirement was resolved to COX-2 by the contrasting effects of the isoform-selective inhibitors SC-560 and NS-398, with the specificity of the latter confirmed by rescue with exogenous PGE_2_. The agreement among these independent manipulations makes an off-target explanation for any individual step unlikely.

The final step of the cascade, the acceleration of anterograde IFT downstream of cAMP, was inferred from a previous study (Jin et al., 2014). This acceleration may be relevant for neural development. The primary cilium is a signaling antenna required for neuronal maturation, and the disruption or removal of neuronal cilia impairs dendritic growth, circuit integration, migration, and cortical circuit formation (Guadiana et al., 2013; Kumamoto et al., 2012; Higginbotham et al., 2012; 2013). By accelerating the elongation of this antenna, DHA may enhance these cilium-dependent processes, providing a subcellular basis for the reported developmental benefits of DHA.

However, this interpretation requires caution. Ciliary length and signaling output are not related in a single, uniform way; four distinct relationships have been proposed, ranging from length changes having no consequence to skewing signaling toward particular pathways (Macarelli et al., 2023). To determine where our system falls, we turned to Hedgehog signaling, the pathway whose dependence on the primary cilium is best established: Smoothened and Gli transcription factors must traffic through the ciliary compartment for the signal to be transduced, and loss of ciliary integrity attenuates Hedgehog output (Goetz and Anderson, 2010; Bangs and Anderson, 2017). Therefore, Hedgehog activity provides the most direct measure of whether a change in ciliary length alters ciliary signaling. We treated neurons with DHA and subsequently stimulated the pathway with recombinant Shh-N or the Smoothened agonist SAG and quantified the expression of the canonical Gli target gene *Ptch1*. Both agonists induced *Ptch1*, but the magnitude of induction was comparable between DHA-treated and control neurons (data not shown). This argues against a simple rheostat relationship for Shh signaling in these neurons, and we consider the altered composition model to be the more likely interpretation. The cascade described here converges on EP4 dependent elevation of cAMP, and cAMP–PKA activity promotes the processing of Gli2 and Gli3 into their repressor forms (Tuson et al., 2011; Niewiadomski et al., 2014). Elongation driven by a Gαs-coupled receptor would therefore be expected to strengthen cAMP-dependent signaling while leaving Gli-dependent transcription unchanged, rather than amplifying all ciliary pathways in parallel.

Ciliary length is also maintained homeostatically, and excessive elongation may reflect a breakdown of length control rather than a physiological response. Overexpression of ciliary GPCRs, such as 5-HT6, produces abnormally long (greater than 30 μm) and frequently branched cilia (Guadiana et al., 2013). The increase we observed was less than twofold (Figure 1), well within the physiological range, and clearly distinct from pathological elongation. Therefore, we regard this as an accelerated developmental elongation rather than a loss of length homeostasis.

Another point concerns the relationship between intracellular Ca^2+^ and ciliary length. In renal cells, a fall in Ca^2+^ or a rise in cAMP elongates cilia (Besschetnova et al., 2010), which at first appears to conflict with our model, in which the Ca^2+^ increase evoked by DHA and GPR40 promotes cilium elongation. This discrepancy can be reconciled at the effector level. In these cells, elongation is driven by cAMP and PKA rather than by low Ca^2+^. Because these cells rely on Ca^2+^-inhibited adenylyl cyclases AC5 and AC6, a decrease in Ca^2+^ levels raises cAMP levels. In contrast, in our model, the relevant cAMP arises from PGE_2_ acting on the ciliary EP4. Therefore, the two systems converge on a common cAMP-dependent effector but differ in how cAMP is generated. The rise in Ca^2+^ lies upstream of this convergence point and activates calcineurin and NFAT-dependent COX-2 induction rather than adenylyl cyclase. Thus, the opposite sign of the Ca^2+^ requirement reflects a difference in upstream wiring rather than a contradiction at the effector. Consistent with this, changes in ciliary length in renal cells were acute (within 3 h) and transcription-independent, whereas the changes we observed developed over several days and required NFAT-dependent COX-2 induction (Figure 6). The calcineurin/NFAT module is well-suited to link the Ca^2+^ signal evoked by Gq-coupled GPR40 to transcription, as NFAT decodes the temporal pattern of Ca^2+^ signals (Tomida et al., 2003).

## 4 Limitations

Several limitations of this study should be acknowledged. First, the functional consequences of DHA-induced ciliary elongation remain undefined. As noted above, our data do not discriminate between the models relating ciliary length to signaling. The analysis of Hedgehog signaling was based solely on the Gli target gene, *Ptch1*. Consequently, the conclusions drawn are limited to this specific gene, as other potential target genes were not investigated in this study. The link between ciliary length and Shh output has been characterized largely in proliferating cells (Ansari et al., 2024), whereas the primary cilia of postmitotic neurons are enriched with neuromodulatory GPCRs, such as SSTR3, MCHR1, and 5-HT6 (Berbari et al., 2008; Guadiana et al., 2013). Therefore, the consequences of elongation may instead lie in signaling through other ciliary receptors. Whether elongation influences neurite outgrowth or synapse formation, both of which depend on an intact cilium (Guadiana et al., 2013; Kumamoto et al., 2012; Higginbotham et al., 2012), remains untested.

Second, we identified a requirement for adenylyl cyclase activity but not for a particular isoform, since SQ22536 acts without isoform selectivity. AC3 is the most likely candidate, as it is the predominant adenylyl cyclase in neuronal primary cilia and is concentrated to a degree that has made it a standard marker of the organelle (Bishop et al., 2007; Berbari et al., 2007). Testing this genetically was not possible here because AC3 immunoreactivity is the signal by which we detect the cilium, and its depletion would remove the readout itself. Resolving which isoform operates downstream of EP4 will require a different labeling strategy.

Third, although calcineurin/NFAT and COX-2 activities are required for ciliary elongation, the transcriptional step linking them has not been resolved. INCA-6 disrupts the interaction between calcineurin and the NFAT family as a whole; therefore, the isoform involved was not identified, and we did not establish that NFAT acts directly at the *Ptgs2* promoter in these neurons.

Fourth, the genetic validation of the receptor was restricted to cultured neurons. The *in vivo* requirement for GPR40 was established pharmacologically using DC260126, and *Ffar1* deficient or conditionally deleted mice were not examined. The *in vivo* experiments also rested on a single acute administration at E11.5 and a single analysis time point at E13.5; therefore, neither the time course nor the persistence of the effect in the developing brain was determined.

Finally, we did not measure fetal brain fatty acid composition in our animals; therefore, the magnitude of the change in fetal brain DHA achieved here is not known directly. However, this effect is well documented for the manipulations used in this study. In C57BL/6 mice, a maternal diet high in n-3 PUFA raised fetal brain DHA by embryonic day 12.5, within the E7.5 to E13.5 window of the present study (Akerele and Cheema, 2020), and the fetal brain is more responsive than the maternal brain to short-term changes in maternal supply (van Goor et al., 2008). In rats, a DHA-enriched maternal diet restored fetal brain DHA in n-3 deficient embryos within 48 h (Schiefermeier and Yavin, 2002), the same interval that separates administration and analysis in our acute experiments. Therefore, a direct measurement would be expected to confirm a well-characterized effect rather than resolve an open question. Nonetheless, we did not quantify the concentrations of DC260126 or GW9508 reaching the fetal brain; therefore, an action mediated through the dam or placenta cannot be formally excluded. However, both compounds produced the same directional effects on dissociated hippocampal neurons, where no maternal or placental compartment exists, arguing against an indirect mechanism.

## 5 Future directions

This study identifies primary cilium elongation as a novel physiological action of DHA. Because primary cilia act as antennae for extracellular signals, ciliary length may influence signaling capacity by changing the membrane surface available for receptors and downstream effectors, thereby altering cilia-dependent signal transduction. This is consistent with the short, signaling poor cilia produced by ciliary assembly mutations in ciliopathies (Hildebrandt et al., 2011; Reiter and Leroux, 2017).

An important question is which ciliary pathways are affected by this elongation of the cilia. Shh signaling engages EP4 and cAMP signaling to maintain IFT and ciliary length, with the loss of EP4 shortening neuroepithelial cilia and impairing neural tube patterning *in vivo* (Ansari et al., 2024). However, the transcriptional output is only one readout of Shh competence. A more direct assessment of the ciliary Shh machinery, for example, by quantifying the ciliary accumulation of Smoothened and GLI proteins, would establish whether cilium elongation alters the capacity of the cilium to transduce Shh, even when target gene expression is unaffected. Confirming the inferred final step, the acceleration of anterograde IFT, remains a goal.

Resolving these questions should clarify the role of DHA in neural circuit formation, adult neurogenesis, and prevention of neurological diseases. Given that defects in ciliary signaling accompany many neurodevelopmental and neuropsychiatric disorders (Guemez-Gamboa et al., 2014; Valente et al., 2014), a DHA-driven mechanism that supports cilium-dependent signaling may provide a basis for therapeutic and preventive strategies.

## 6 Materials and methods

### 6.1 Primary culture of mouse hippocampal neurons

Primary hippocampal neurons were isolated from E16.5 wild-type ICR mouse embryos (Japan SLC and CLEA Japan). Hippocampi were dissected in ice-cold phosphate-buffered saline (PBS) containing 0.4% glucose and digested with papain (Nacalai Tesque) and DNase I (Sigma-Aldrich) for 20 min at 37 °C. The tissue was gently triturated with a fire-polished Pasteur pipette, and the dissociated cells were collected by centrifugation. The cell pellet was resuspended in Neurobasal medium (Thermo Fisher Scientific) supplemented with 10% fetal bovine serum (FBS; Japan Bioserum) and plated onto 13-mm glass coverslips (Matsunami) or 35-mm dishes (Greiner) precoated with 0.1 mg/mL poly-D-lysine (Sigma-Aldrich). After 2–3 h, the plating medium was replaced with Neurobasal medium supplemented with 2% B-27 and 1% GlutaMAX (Thermo Fisher Scientific). For cultures used in Western blotting and RT-qPCR, cytosine β-D-arabinofuranoside (Sigma-Aldrich) was added to a final concentration of 10 μM to suppress the proliferation of non-neuronal cells. Neurons were maintained at 37 °C in a humidified atmosphere of 5% CO_2_.

### 6.2 Chemicals and reagents

All compounds were dissolved in dimethyl sulfoxide (DMSO) to prepare stock solutions, as detailed in Table 1. Stock solutions were aliquoted and stored at −30 °C. Before use, stocks were diluted in culture medium such that the final DMSO concentration did not exceed 0.25% (v/v). Control cultures received an equivalent volume of DMSO as a vehicle control.

**TABLE 1:** Compounds used in this study, with suppliers, catalogue numbers, working concentrations, molecular targets, and the figures in which each was used.

Compound	Manufacturer	Item no.	Working conc.	Target (action)	Used in
DHA	Nacalai Tesque	14122-64	1–10 μM	GPR40 agonist	Figures 1, 3A,B,G,H, 5A–C, 6A,B, 7, 8A,F,G; Supplementary Figures S3, S4
DHA	Cayman Chemical	90310	50 mg/kg, i.p.	GPR40 agonist	Figures 3C,D
DC260126	Sigma-Aldrich	SML1050	10 μM	GPR40 antagonist	Figures 3A,B
DC260126	Sigma-Aldrich	SML1050	50 mg/kg, i.p.	GPR40 antagonist	Figures 3C,D
TAK-875	AdooQ BioScience	A11018	25 μM	GPR40 agonist	Figures 4A,B, 7B,C
GW9508	Cayman Chemical	10008907	25 μM	GPR40 agonist	Figures 4A,B, 5D,E, 6C,D, 7B,C,F,G, 8D,E
GW9508	Cayman Chemical	10008907	50 mg/kg, i.p.	GPR40 agonist	Figures 4C,D
Cyclosporine A	Merck Millipore	30024	200 nM	Calcineurin inhibitor	Figures 5B–E
FK-506	Cayman Chemical	10007965	10 μM	Calcineurin inhibitor	Figures 5B–E
INCA-6	Tocris Bioscience	2162	1 μM	Calcineurin–NFAT interaction inhibitor	Figures 6A–D
SC-560	Cayman Chemical	70340	5 μM	COX-1 inhibitor	Figures 7D–G
NS-398	Cayman Chemical	70590	5 μM	COX-2 inhibitor	Figures 7D–G, 8F,G
Prostaglandin E_2_	Nacalai Tesque	29334-34	2, 20, 200 pg/mL	EP receptor agonist	Figures 8B,C,F,G
AH23848	Cayman Chemicall	19023	10 μM	EP4 antagonist	Figures 8D,E
G3335	Santa Cruz Biotechnology	sc-202771	30 μM	PPARγ antagonist	Supplementary Figure S3
GW9662	Santa Cruz Biotechnology	sc-202641	10 μM	PPARγ antagonist	Supplementary Figure S3
SQ22536	Tokyo Chemical Industry	Q0105	100 μM	Adenylyl cyclase inhibitor	Supplementary Figure S4

### 6.3 Immunocytochemistry

Cells grown on poly-D-lysine–coated glass coverslips were fixed in 3.7% formaldehyde in Krebs’ buffer (10 mM HEPES-NaOH [pH 7.0], 118 mM NaCl, 4.7 mM KCl, 1.2 mM KH_2_PO_4_, 1.2 mM MgSO_4_, 4.2 mM NaHCO_3_, 2 mM CaCl_2_, 10 mM glucose, 400 mM sucrose) on ice for 15 min. After three washes in 1× PBS, cells were permeabilized with 0.05% Triton X-100 in PBS on ice for 10 min and blocked with 10% FBS in 0.05% Triton X-100/PBS for 30 min at room temperature. Coverslips were incubated with primary antibodies (Table 2) diluted in PBS containing 0.1% Triton X-100 and 10% FBS overnight at 4°C. After three washes with PBS, coverslips were incubated with fluorophore-conjugated secondary antibodies (Table 2) in PBS for 40 min at room temperature in the dark. After three further washes with PBS, coverslips were mounted on glass slides (Matsunami) in 50% glycerol/PBS and sealed with nail polish. Fluorescence images were acquired on an Axioplan 2 microscope (Carl Zeiss) equipped with a Plan-Apochromat 63×/1.40 NA oil-immersion objective and AxioVision 3 software (Carl Zeiss), or on an EVOS microscope (Thermo Fisher Scientific) with a Plan-Apochromat 63×/1.40 NA oil-immersion objective or on a Mica WideFocal system (Leica Microsystems) equipped with a 63×/1.20 NA water-immersion objective. Primary cilium length was measured using Fiji (NIH).

**TABLE 2:** Primary and secondary antibodies used in this study.

Antibody	Host/clonality	Supplier	Cat. no.	RRID	Dilution
Primary antibodies					
Anti-COX-2	Rabbit, polyclonal	Cell Signaling Technology	4842	RRID:AB_2084968	1:250
Anti-β-actin (clone 4C2)	Mouse, monoclonal	Merck Millipore	MABT825	RRID:AB_2571580	1:5,000
Anti-AC3 (ADCY3, clone C-20)#	Rabbit, polyclonal	Santa Cruz Biotechnology	sc-588	RRID:AB_630839	1:250
Anti-ARL13B (clone N295B/66)	Mouse, monoclonal	NeuroMab	75-287	RRID:AB_2341543	1:5,000
Anti-β-tubulin (clone 10G10)	Mouse, monoclonal	Fujifilm	014-25041	RRID:AB_3717508	1:3000
Secondary antibodies					
HRP-conjugated anti-rabbit IgG	Donkey	Amersham/Cytiva	NA934	RRID:AB_772206	1:2,000
HRP-conjugated anti-mouse IgG	Goat	Bio-Rad	1706516	RRID:AB_2921252	1:5,000
Alexa Fluor 594 anti-mouse IgG	Goat	Thermo Fisher Scientific	A-11032	RRID:AB_2534091	1:1,000
Alexa Fluor 488 anti-rabbit IgG	Goat	Thermo Fisher Scientific	A-11008	RRID:AB_143165	1:1,000

^#^ The anti-AC3 antibody (sc-588) was discontinued by the manufacturer.

### 6.4 Plasmid construction

The full-length coding sequence of mouse GPR40 was amplified from C57BL/6 mouse genomic DNA, which served as the template because the GPR40 coding sequence is located within a single exon. The primers used were as follows: forward, 5′-GGA​A*CTC​GAG*GCC​ACC**ATG**GAC​CTG​CCC​CCA​CAG​CTC-3′; reverse, 5′-CCT​T*GAA​TTC*CTT​CTG​AAT​TGT​TCC​TCT​TTG​AG-3′. The forward primer contained an *Xho*I site (italic) followed by a Kozak consensus sequence (GCCACC) immediately upstream of the initiation codon (bold), and the reverse primer contained an *Eco*RI site (italic) and omitted the stop codon to allow in-frame fusion with the downstream EGFP coding sequence. Amplification was performed using KOD-Plus-Neo DNA polymerase (Toyobo) according to the manufacturer’s instructions. The purified product was digested with *Xho*I and *Eco*RI and ligated into the corresponding sites of pEGFP-N2 (Clontech), yielding a construct encoding full-length mouse GPR40 fused to EGFP at the C-terminus (GPR40-EGFP). The insert sequence and reading frame across both cloning junctions were verified by Sanger sequencing.

### 6.5 RNAi

RNAi constructs targeting mouse *Ffar1* were designed using the BLOCK-iT RNAi Designer and cloned into pcDNA6.2-GW/EmGFP-miR (BLOCK-iT Pol II miR RNAi Expression Vector Kit; Thermo Fisher Scientific) according to the manufacturer’s instructions. The target sequences were 5′-TGG​GCT​TTC​CAT​TGA​ACT​TGT-3′ (RNAi #1) and 5′-TCC​AAT​GTG​GCT​AGT​TTC​ATA-3′ (RNAi #2), corresponding to nucleotides 53–73 and 727–747 of the mouse *Ffar1* coding sequence (GenBank: AB095745.1), respectively. The non-targeting pcDNA6.2-GW/EmGFP-miR-neg plasmid was used as a negative control. Because EmGFP and the RNAi cassette were transcribed as a single transcript, transfected cells were identified by EmGFP fluorescence. Knockdown efficiency was validated in HEK293T cells co-transfected with GPR40-EGFP and either the control or one of the two *Ffar1* RNAi vectors using PEI MAX (Polysciences). Cells were lysed in 1× RIPA buffer 48 h after transfection, protein concentrations were determined using a BCA protein assay kit (Fujifilm Wako Pure Chemical), and 15 μg of protein per lane was resolved by SDS-PAGE. Immunoblotting was performed using anti-GFP (MBL) and anti-β-tubulin (Fujifilm Wako Pure Chemical) antibodies, and signals were detected using a ChemiDoc MP imaging system (Bio-Rad). This heterologous system was used because the low transfection efficiency of primary hippocampal neurons precluded the population-level assessment of knockdown.

Dissociated hippocampal neurons were transfected by electroporation using the Neon Transfection System (Thermo Fisher Scientific). Briefly, 5 μg of plasmid DNA was introduced into 1 × 10^6^ cells immediately after dissociation, and cells were plated onto glass coverslips and maintained as described above. DHA (5 μM) was added 24 h after plating, and the neurons were fixed 24 h later, immunostained for AC3, and cilium length was measured in EmGFP-positive cells.

### 6.6 Protein extraction from cultured neurons

Hippocampal neurons (1.0 × 10^6^ cells) cultured in 35-mm poly-D-lysine–coated dishes (Greiner Bio-One) were washed twice with ice-cold PBS and lysed in 120 μL of RIPA buffer (20 mM Tris-HCl [pH 8.0], 150 mM NaCl, 1 mM EDTA, 1% Triton X-100, 0.1% SDS, 0.1% sodium deoxycholate) supplemented with protease and phosphatase inhibitors (1 mM DTT, 1 mM PMSF, 10 μM leupeptin, 10 mM NaF, 10 mM β-glycerophosphate) on ice for 5 min. Lysates were collected with a cell scraper, transferred to microcentrifuge tubes, and centrifuged at 15,000 × *g* for 10 min at 4 °C. Supernatants were mixed with 5× SDS sample buffer (250 mM Tris-HCl [pH 6.8], 10% SDS, 500 mM DTT, 50% glycerol, 0.2% bromophenol blue) and heated at 95 °C for 5 min before SDS-PAGE.

### 6.7 Western blot analysis

Protein samples were resolved by SDS-PAGE and transferred onto PVDF membranes (Millipore) using a Trans-Blot SD Semi-Dry Transfer Cell (Bio-Rad) at 2 mA/cm^2^ for 90 min at room temperature. Membranes were blocked with 3% skim milk (Morinaga) in TBST (20 mM Tris-HCl [pH 7.4], 150 mM NaCl, 0.05% Tween-20) for 1 h at room temperature and incubated overnight at 4 °C with primary antibodies (Table 2) diluted in 3% skim milk/TBST. Membranes were washed three times (8 min each) with TBST and incubated with horseradish peroxidase (HRP)-conjugated secondary antibodies in 1% skim milk/TBST for 1 h at room temperature. After three further washes in TBST (8 min each), immunoreactive bands were detected with ECL reagent (GE Healthcare), visualized on X-ray film (Fujifilm), and quantified using Fiji (NIH).

### 6.8 Real-time quantitative PCR (RT-qPCR)

Total RNA was isolated from neurons cultured in 35-mm dishes using TRIzol reagent (Thermo Fisher Scientific) according to the manufacturer’s instructions. For the time-course analysis of cyclooxygenase expression, DHA (5 μM) was added at DIV2, and RNA was harvested at 0, 6, and 12 h. RNA concentration and purity were assessed spectrophotometrically with a Q5000 instrument (TOMY). RT-qPCR was performed in technical triplicate on a StepOne Real-Time PCR System (Thermo Fisher Scientific) using the Luna Universal One-Step RT-qPCR Kit (New England Biolabs) according to the manufacturer’s protocol. Each 20-μL reaction contained 40 ng of total RNA. Reverse transcription was performed at 55 °C for 10 min, followed by 40 cycles (95 °C for 10 s; 64 °C for 1 min). Amplification specificity was confirmed by melting-curve analysis. The relative expression of *Ptgs1* (COX-1) and *Ptgs2* (COX-2) was normalized to *Gapdh* and calculated using the comparative CT (2^(−ΔΔCT)) method. Primer sequences are provided in Table 3.

**TABLE 3:** Primer sequences used for RT-qPCR.

Gene	Forward (5′–3′)	Reverse (5′–3′)	RefSeq mRNA	Gene ID
*Gapdh*	AAA​TGG​TGA​AGG​TCG​GTG​TG	TGAAGGGGTCGTTGATGG	NM_008084	14433
*Ptgs1*	GAT​TGT​ACT​CGC​ACG​GGC​TAC	GGA​TAA​GGT​TGG​ACC​GCA​CT	NM_008969	19224
*Ptgs2*	GTG​TGC​GAC​ATA​CTC​AAG​CAG​GA	TGA​AGT​GGT​AAC​CGC​TCA​GGT​G	NM_011198	19225

### 6.9 Enzyme-linked immunosorbent assay (ELISA)

PGE_2_ levels in the culture medium were quantified using a Prostaglandin E2 ELISA Kit (Abcam), according to the manufacturer’s protocol. Hippocampal neurons were cultured for 33 h, after which the medium was replaced with Neurobasal medium supplemented with 1 mM glutamine for serum starvation. After 3 h, DHA was added to a final concentration of 5 μM, and culture supernatants were collected 0, 12, and 24 h later by centrifugation at 15,000 × *g* for 1 min at room temperature. Absorbance was measured at 405 nm using a TriStar2 LB942 microplate reader (Berthold Technologies), and PGE_2_ concentrations were determined by interpolation from a standard curve.

### 6.10 Calcium imaging

Cultured hippocampal neurons were transfected with a plasmid encoding the genetically encoded calcium indicator jRGECO1a (pGP-CMV-NES-jRGECO1a; Addgene #61563) by electroporation using the Nucleofector system (Lonza, program “O-05”) according to the manufacturer’s instructions. Transfected cells were seeded onto poly-D-lysine–coated glass-bottom dishes and maintained in Neurobasal medium supplemented with 2% B-27, 1 mM glutamine, and penicillin/streptomycin at 37 °C under 5% CO_2_. Forty-six hours after transfection, the medium was replaced with 1× HBSS (Thermo Fisher Scientific), and cells were incubated for an additional 2 h at 37 °C. Live-cell calcium imaging was performed on an inverted fluorescence microscope (IX81; Olympus) equipped with a UPlanFL N 10×/0.30 Ph1 objective (Olympus) and controlled by MetaMorph software (Molecular Devices). DHA (final concentration, 5 μM) was applied to the imaging medium, and fluorescence images were acquired at 3-s intervals. Fluorescence intensities were quantified using Fiji (NIH), and pseudocolored images were generated using the Royal lookup table.

### 6.11 Animals and diets

All animal procedures were conducted in accordance with the guidelines of Kwansei Gakuin University and were approved by its Institutional Animal Care and Use Committee (approval nos. 2019-26 and 2021-15) and by the Animal Care and Use Committee of Tokushima Bunri University (approval no. KP25-73-1). Mice were obtained from Japan SLC (Hamamatsu, Japan) and maintained under a 12-h light/dark cycle at constant temperature (22 °C–25 °C) and humidity, with food and water available *ad libitum*. The morning on which a vaginal plug was detected was designated embryonic day 0.5 (E0.5). Pregnant dams were euthanized by cervical dislocation on E13.5, and fetal brains were rapidly dissected for subsequent analyses.

Three custom diets based on the AIN-93G formulation (Reeves et al., 1993) were prepared by Oriental Bio Service (Kyoto, Japan): a control diet (Control; AIN-93G) containing 7% (w/w) soybean oil; an n-3–deficient diet (low n-3), in which soybean oil was replaced with 7% corn oil; and an n-3–enriched diet (high n-3) containing 4% corn oil supplemented with 3% DHA oil. The full composition is given in Table 4. The three diets were isocaloric (368 kcal/100 g) and matched for total fat (7% oil), differing only in fatty acid composition. To minimize lipid peroxidation, diets were divided into single-use aliquots, stored at −30 °C in the dark, and provided fresh every day.

**TABLE 4:** Compositions of experimental diets.

Ingredient composition ingredient (%w/w)	Control diet (AIN-93G)	Low n-3 diet	DHA-rich diet
Casein	20.00	20.00	20.00
L-cystine	0.30	0.30	0.30
Cornstarch	39.75	39.75	39.75
Pre-gelatinized cornstarch	13.20	13.20	13.20
Sucrose	10.00	10.00	10.00
Soybean oil	7.00	—	—
Corn oil	—	7.00	4.00
DHA oil	—	—	3.00
Cellulose powder	5.00	5.00	5.00
AIN-93G mineral mix	3.50	3.50	3.50
AIN-93G vitamin mix	1.00	1.00	1.00
Choline bitartrate	0.25	0.25	0.25
tert-Butylhydroquinone (TBHQ)	0.0014	0.0014	0.0014
Total	100.00	100.00	100.00

Proximate nutrient composition (per 100 g diet)	Control diet (AIN-93G)	Low n-3 diet	DHA-rich diet
Moisture (g)	9.00	9.00	9.00
Crude protein (g)	18.07	18.07	18.07
Crude fat (g)	7.26	7.26	7.26
Crude ash (g)	3.08	3.08	3.08
Crude fiber (g)	5.00	5.00	5.00
Nitrogen-free extract (NFE) (g)	57.60	57.60	57.60
Total energy (kcal)	368.0	368.0	368.0

Fatty acid composition#	Control diet (AIN-93G)	Low n-3 diet	DHA-rich diet
Myristic acid (14:0)	0.01	0.00	0.00
Pentadecanoic acid (15:0)	0.00	0.00	0.00
Palmitic acid (16:0)	0.74	0.79	0.45
Palmitoleic acid (16:1n-7)	0.01	0.01	0.00
Stearic acid (18:0)	0.30	0.14	0.08
Oleic acid (18:1n-9)	1.65	2.09	1.19
Linoleic acid (LA, 18:2n-6)	3.75	3.84	2.20
α-Linolenic acid (ALA, 18:3n-3)	0.46	0.06	0.03
Arachidic acid (20:0)	0.03	0.03	0.02
Eicosenoic acid (20:1n-9)	0.01	0.02	0.01
Behenic acid (22:0)	0.03	0.01	0.00
Docosapentaenoic acid (DPA, 22:5n-3)	0.00	0.00	0.00
Docosahexaenoic acid (DHA, 22:6n-3)	0.00	0.00	3.00
Lignoceric acid (24:0)	0.01	0.01	0.01
Others	0.01	0.01	0.00
Saturated fatty acids (SFA)	1.11	0.98	0.56
Monounsaturated fatty acids (MUFA)	1.67	2.11	1.21
Polyunsaturated fatty acids (PUFA)	4.21	3.90	5.23
Total n-3 PUFA	0.46	0.06	3.03
Total n-6 PUFA	3.75	3.84	2.2

# Fatty acid composition is shown as percentage of total diet weight (g/100 g, w/w).

### 6.12 *In vivo* administration of DHA and GPR40 ligands

Two independent *in vivo* paradigms were used. Dietary manipulation. Pregnant dams were randomly assigned to one of the three diets described above on E7.5 (n = 5–6 dams per group) and maintained on the assigned diet until analysis at E13.5. Acute pharmacological administration. DHA (50 mg/kg), the GPR40 antagonist DC260126 (50 mg/kg), or the GPR40 agonist GW9508 (50 mg/kg), alone or in combination as indicated, was administered to pregnant dams by intraperitoneal injection on E11.5. Dams in this paradigm were maintained on standard chow. Embryonic brains were collected at E13.5.

### 6.13 Immunohistochemistry

Heads dissected from E13.5 mouse embryos were fixed in 4% paraformaldehyde (PFA; Fujifilm) at 4 °C overnight. After three washes with PBS, tissues were cryoprotected by sequential immersion in 10% and 20% sucrose in PBS at 4 °C until they sank, embedded in OCT compound (Sakura Finetek), and snap-frozen in isopentane (Nacalai Tesque). Cryosections (12 μm) were prepared on a cryostat (NX70; Micro-edge Instruments) and mounted on MAS-coated glass slides (Matsunami). Sections were blocked with 10% normal goat serum (Vector Laboratories) in PBS containing 0.3% Triton X-100 (PBT) at room temperature for 1 h and incubated with anti-ARL13B primary antibody (Table 2) diluted in blocking solution at 4 °C overnight. After three washes with PBT, sections were incubated with Alexa Fluor 594–conjugated anti-mouse IgG secondary antibody (1:1,000; Thermo Fisher Scientific) in blocking solution at room temperature for 1 h. After three further washes with PBT, nuclei were counterstained with 4′,6-diamidino-2-phenylindole (DAPI; Roche; 1:5,000) for 5 min. Sections were mounted in 50% glycerol in PBS and coverslipped (Matsunami). Fluorescence images were acquired on an LSM700 confocal microscope (Carl Zeiss) with a Plan-Apochromat 63×/1.40 NA oil-immersion objective using ZEN 2009 software (Carl Zeiss). Image processing was performed in Fiji (NIH).

### 6.14 Quantification of primary cilium length

Primary cilium length was quantified using Fiji. Primary cilia were detected by immunostaining for AC3 in cultured neurons and for ARL13B in brain sections. In tissue sections, measurements were restricted to the primary cilia of cortical neurons in the preplate of the cerebral cortex, identified on the basis of their laminar position and nuclear morphology. The length of each cilium was determined by manually tracing its longitudinal axis with the segmented-line tool in Fiji. A subset of the data was independently measured by an investigator blinded to the experimental conditions.

### 6.15 Statistical analysis

All experiments were performed independently at least three times. Data are presented as mean ± standard error of the mean (SEM) unless otherwise stated. Statistical analyses were performed using Microsoft Excel (Microsoft) and GraphPad Prism version 8.0.2 (GraphPad Software). The normality of each dataset was assessed with the D'Agostino–Pearson omnibus test before selecting a test. For comparisons between two groups, a two-tailed Student’s t-test was used when the data were normally distributed, and the Mann–Whitney *U* test when they were not. For comparisons among three or more groups with a single factor, one-way analysis of variance (ANOVA) was used, followed by Dunnett’s test when all groups were compared with a single control and by Tukey’s test when all pairwise comparisons were of interest. For experiments with two factors (e.g., treatment × inhibitor), two-way ANOVA followed by Tukey’s *post hoc* test was used. The test applied to each dataset is specified in the corresponding figure legend. A *p* value of less than 0.05 was considered statistically significant. Significance was indicated in the figures as *p < 0.05, **p < 0.01, ***p < 0.001, and ****p < 0.0001, with ns denoting not significant.

## Data Availability

The original contributions presented in the study are included in the article/Supplementary Material, further inquiries can be directed to the corresponding author.
