# Supplementary material for: DHA elongates the neuronal primary cilium through GPR40-mediated signaling
Source: Front Cell Dev Biol. 2026 Sep 29;14:1910684. doi: 10.3389/fcell.2026.1910684 (PMC13658593; doi:10.3389/fcell.2026.1910684)
Supplement: Supplementary file 1 [file Supplementaryfile1.pdf]

Supplementary Figure 1

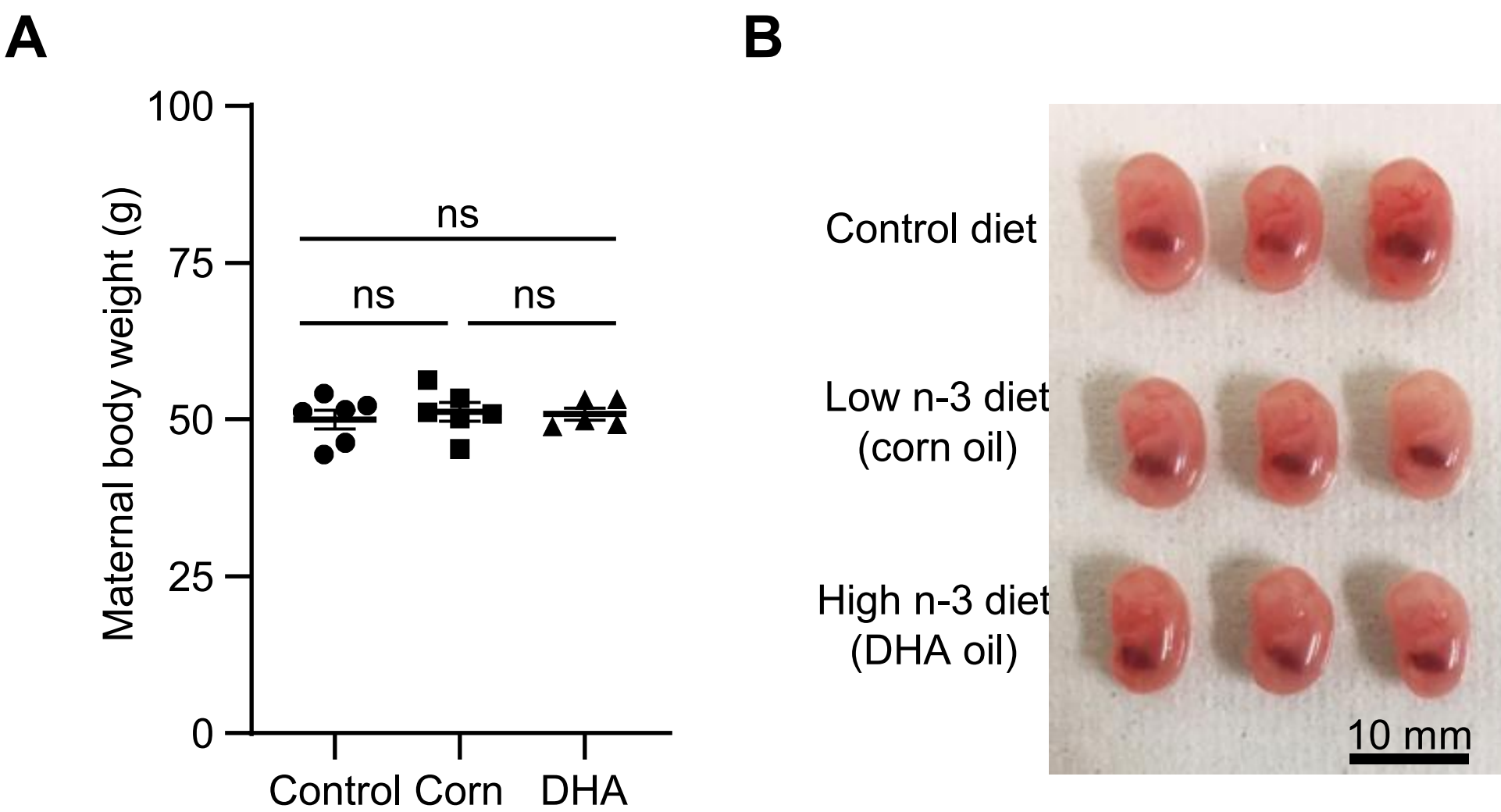

Supplementary Figure 1. Maternal dietary n-3 fatty acid manipulation during pregnancy does not affect maternal body weight or fetal growth.

Pregnant mice were randomly assigned to three groups on gestational day (E) 7.5 and maintained on a control diet (AIN-93G), a low-n-3 diet, or a high-n-3 diet until analysis. (A) Maternal body weight measured on E13.5. No significant difference was observed among the three groups. (B) Representative images of mouse embryos at E13.5. No apparent differences in fetal size or gross morphology were detected among the dietary groups. Data are presented as mean ± SEM (n = 5 dams per group); statistical significance was assessed by one-way ANOVA followed by Tukey's post hoc test. ns, not significant. Scale bar, 10 mm.

Supplementary Figure 2

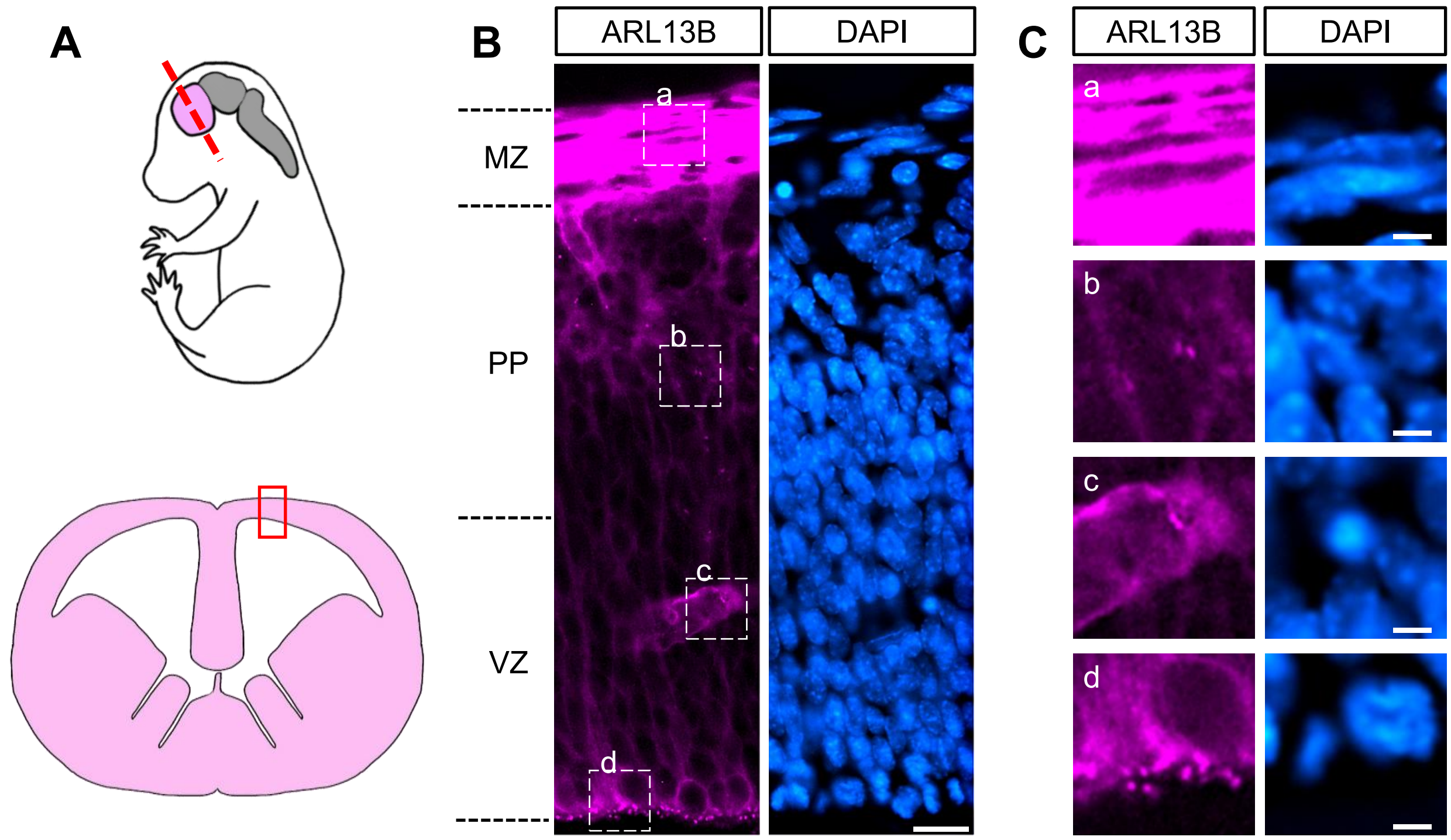

Supplementary Figure 2. Primary cilia in the developing fetal mouse brain visualized by ARL13B immunostaining.

(A) Schematic of a coronal section of the fetal mouse brain, with the analyzed region indicated by the red box. (B) Representative fluorescence image of the boxed region in (A), spanning the marginal zone (MZ), preplate (PP), and ventricular zone (VZ), immunostained for the ciliary marker ARL13B (magenta) and counterstained with DAPI (blue) to label nuclei. Scale bar, 20 µm. (C) Higher-magnification views of the dashed boxes in (B): (a) the region adjacent to the skin and meninges; (b) primary cilia of neurons; (c) primary cilia of vascular endothelial cells; and (d) primary cilia of radial glial cells. Scale bar, 5 µm. In this study, primary cilium length was measured for the neuronal population in region (b).

Supplementary Figure 3

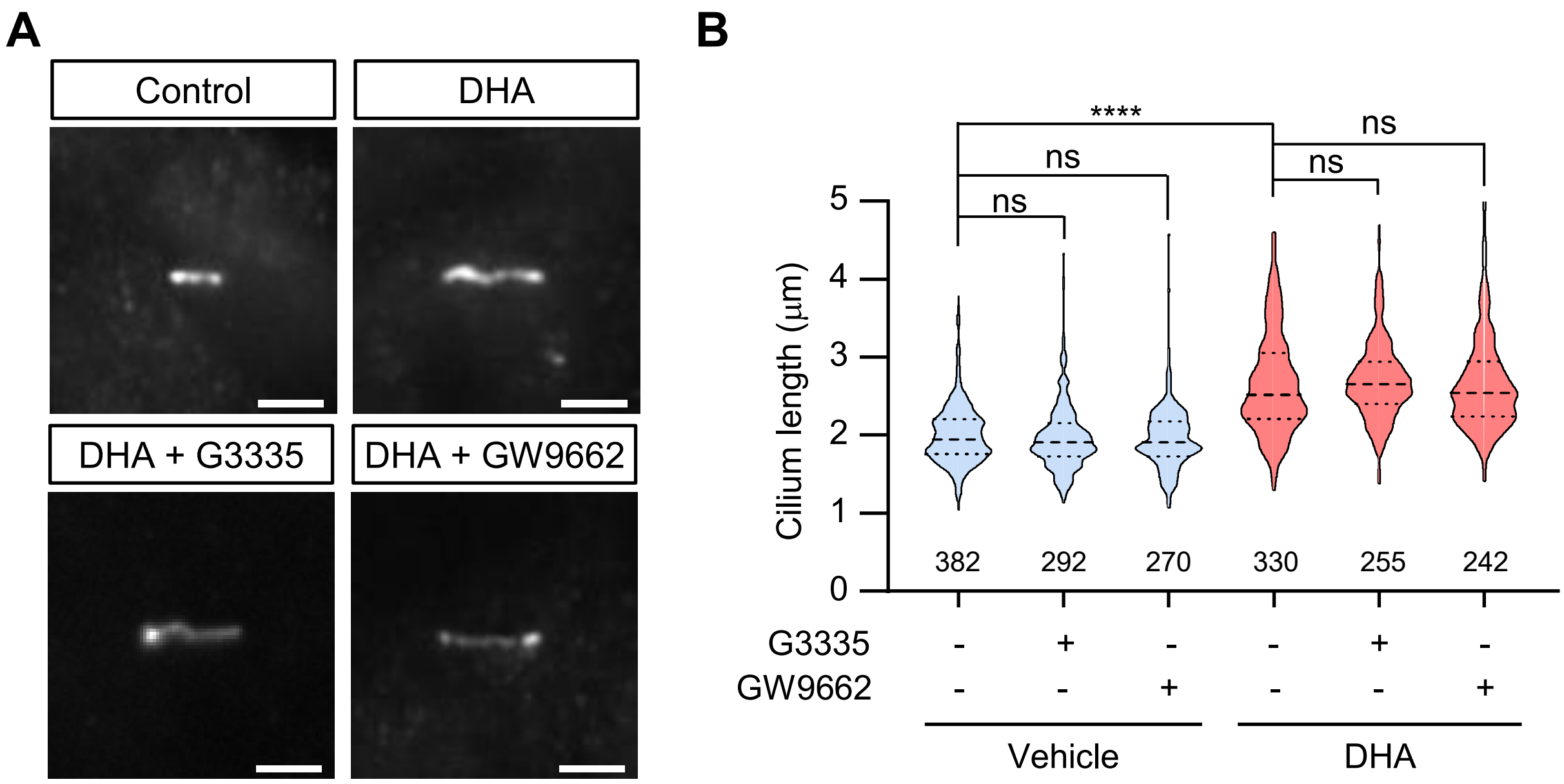

Supplementary Figure 3. DHA-induced primary cilium elongation does not require PPARγ. (A) Representative images of primary mouse hippocampal neurons at 2 days *in vitro* (DIV2) immunostained for adenylyl cyclase III (ACIII), treated with vehicle (Control) or DHA (5 µM) in the absence or presence of the selective PPARγ antagonists G3335 (30 µM) or GW9662 (10 µM). Scale bar, 2 µm. (B) Quantification of primary cilium length. DHA increased cilium length to 1.3-fold of the vehicle control, and this increase was unaffected by either G3335 or GW9662; neither antagonist alone altered cilium length. Violin plots show the distribution of individual cilia; dashed lines indicate the median and quartiles, and the number of cilia analyzed is given above each violin. ****$p < 0.0001$; ns, not significant (two-way ANOVA followed by Tukey's post hoc test).

Supplementary Figure 4

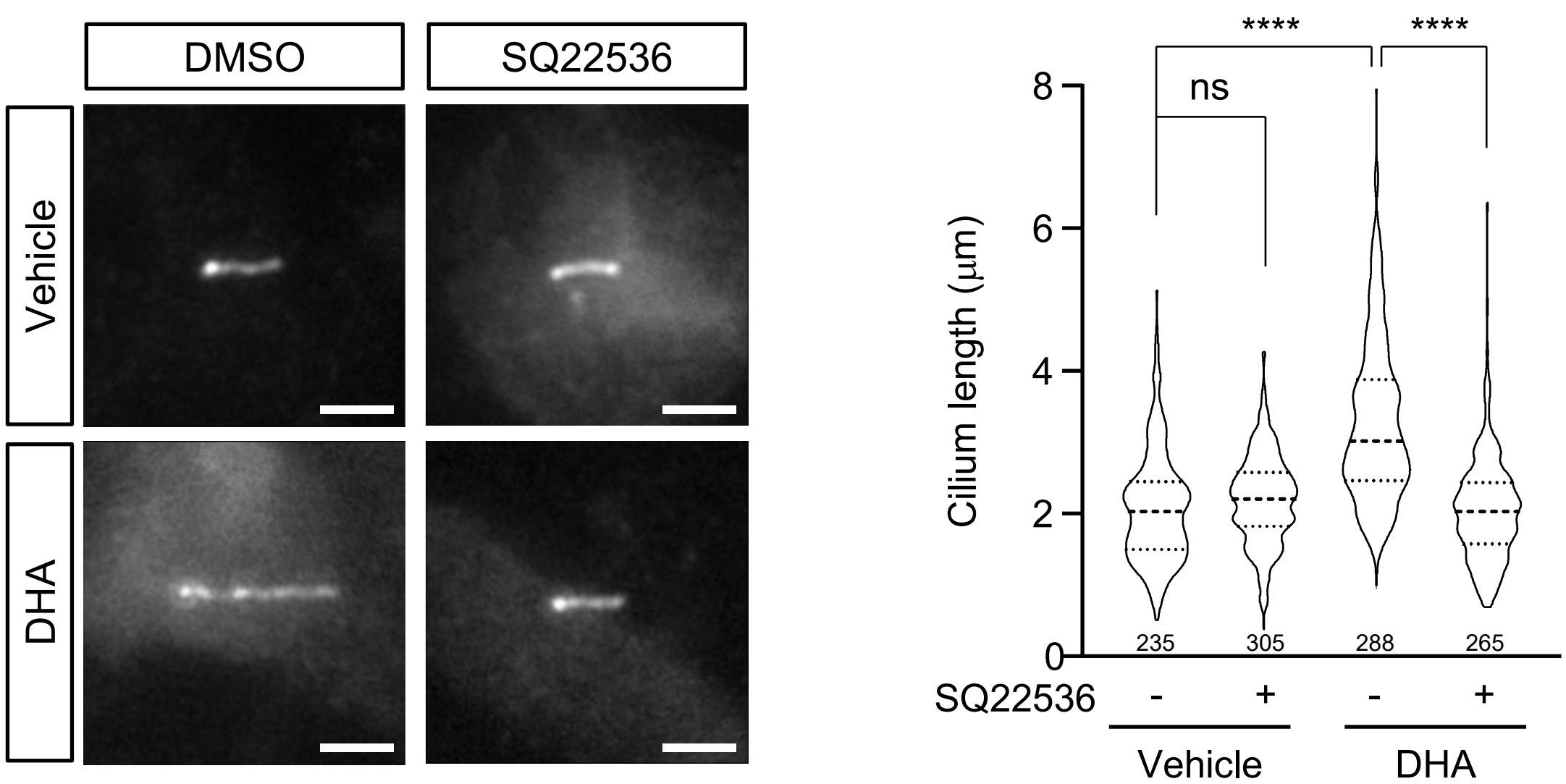

Supplementary Figure4. Adenylyl cyclase activity is required for DHA-induced primary cilium elongation.

Primary mouse hippocampal neurons were co-treated with vehicle (DMSO) or DHA (5 µM) and vehicle (DMSO) or the adenylyl cyclase inhibitor SQ22536 (100 µM) from DIV1 for 24 h, and fixed at DIV2. Cilium length was measured in neurons immunostained for adenylyl cyclase 3 (AC3). (Left) Representative images of primary cilia. Scale bar, 2 µm. (Right) Quantification of cilium length. DHA increased cilium length in the absence of SQ22536, whereas this increase was abolished in the presence of SQ22536. SQ22536 alone did not alter basal cilium length. Violin plots show the distribution of individual cilia, with dashed lines indicating the median and quartiles; the number of cilia analyzed is given above each violin. ****$p < 0.0001$; ns, not significant (two-way ANOVA followed by Tukey's post hoc test; N = 3 independent cultures).
